# Bioluminescence imaging of *Cyp1a1-*luciferase reporter mice demonstrates prolonged activation of the aryl hydrocarbon receptor in the lung

**DOI:** 10.1038/s42003-024-06089-6

**Published:** 2024-04-10

**Authors:** Nicolas Veland, Hannah J. Gleneadie, Karen E. Brown, Alessandro Sardini, Joaquim Pombo, Andrew Dimond, Vanessa Burns, Karen Sarkisyan, Chris Schiering, Zoe Webster, Matthias Merkenschlager, Amanda G. Fisher

**Affiliations:** 1 Epigenetic Memory Group, MRC Laboratory of Medical Sciences, Imperial College London Hammersmith Hospital Campus, Du Cane Road, London, W12 OHS UK; 2 Whole Animal Physiology and Imaging, MRC Laboratory of Medical Sciences, Imperial College London, Hammersmith Hospital Campus, Du Cane Road, London, W12 0HS UK; 3 Senescence Group, MRC Laboratory of Medical Sciences, Imperial College London Hammersmith Hospital Campus, Du Cane Road, London, W12 0HS UK; 4 Department of Biochemistry, University of Oxford, Oxford, OX1 3QU UK; 5 Synthetic Biology Group, MRC Laboratory of Medical Sciences, Imperial College London Hammersmith Hospital Campus, Du Cane Road, London, W12 0HS UK; 6 Inflammation and Obesity Group, MRC Laboratory of Medical Sciences, Imperial College London Hammersmith Hospital Campus, Du Cane Road, London, W12 0HS UK; 7 Transgenics & Embryonic Stem Cell Facility, MRC Laboratory of Medical Sciences, Imperial College London Hammersmith Hospital Campus, Du Cane Road, London, W12 0HS UK; 8 Lymphocyte Development Group, MRC Laboratory of Medical Sciences, Imperial College London Hammersmith Hospital Campus, Du Cane Road, London, W12 0HS UK

**Keywords:** Cellular imaging, Bioluminescence imaging, Mouse

## Abstract

Aryl hydrocarbon receptor (AHR) signalling integrates biological processes that sense and respond to environmental, dietary, and metabolic challenges to ensure tissue homeostasis. AHR is a transcription factor that is inactive in the cytosol but upon encounter with ligand translocates to the nucleus and drives the expression of AHR targets, including genes of the cytochrome P4501 family of enzymes such as *Cyp1a1*. To dynamically visualise AHR activity in vivo, we generated reporter mice in which firefly luciferase (*Fluc*) was non-disruptively targeted into the endogenous *Cyp1a1* locus. Exposure of these animals to FICZ, 3-MC or to dietary I3C induced strong bioluminescence signal and *Cyp1a1* expression in many organs including liver, lung and intestine. Longitudinal studies revealed that AHR activity was surprisingly long-lived in the lung, with sustained *Cyp1a1* expression evident in discrete populations of cells including columnar epithelia around bronchioles. Our data link diet to lung physiology and also reveal the power of bespoke *Cyp1a1-Fluc* reporters to longitudinally monitor AHR activity in vivo.

## Abstract (web summary)

Generation of an endogenous *Cyp1a1* luciferase reporter mouse model to dynamically and longitudinally visualize AHR activity throughout lifespan and in response to ligand exposure, reveals prolonged AHR activation in the lung.

## Introduction

The aryl hydrocarbon receptor regulates cellular physiology and organ homeostasis^1,2^. It was identified in the early 1990s as an environmental-sensor, with structural similarity to the class 1 basic helix-loop-helix-PER-ARNT-SIM (bHLH-PAS) family of transcription factors^3–5^ and subsequently shown to be activated by a range of ligands^6^. AHR recognises external xenobiotics, such as the polycyclic aromatic hydrocarbon dioxin, as well as endogenous metabolites including a plethora of compounds derived from tryptophan and dietary components generated by microbiota and host metabolism^1,7–10^. AHR is maintained in an inactive state in the cytoplasm, supported by a chaperone complex that includes 90 kDa heat shock protein (HSP90), AHR-interacting protein (AIP), co-chaperone p23 and SRC protein kinase. Ligand binding causes AIP to dissociate and triggers conformational changes that lead to the import of the complex into the nucleus where AHR binds to AHR nuclear translocator (ARNT, also known as HIF1β) and drives the expression of multiple target genes^1^. Importantly, AHR activity induces the expression of enzymes of the cytochrome P450 family (*Cyp1a1, Cyp1b1*) which are capable of oxygenating and metabolically degrading endogenous and exogenous high affinity ligands^11–16^. In addition, AHR activity induces expression of the AHR repressor (AHRR), which shares homology with AHR, ARNT and TiParp^17,18^ and competes with the AHR-ligand complex for ARNT binding, thereby creating a negative feedback loop that regulates AHR activation. Finally, AHR also regulates the expression of TiParp which in turn mediates the ribosylation and degradation of AHR^19^. In this setting, interactions between AHR and ligand stimulate *Cyp1a1*, *Ahrr* and *TiParp* expression that subsequently act to degrade AHR ligand, reduce AHR availability, and counter AHR activation. Failures in this self-limiting process that lead to a dysregulated AHR pathway are linked to disease pathology and increased cancer risk^20–36^.

Our appreciation of the importance of AHR signalling in sensing environmental and pathogen exposures, regulating tissue physiology, immune responses, and disease ontogeny, has increased substantially over the last decade. In particular, advances in the metabolic profiling of dietary response^37,38^, single-cell transcriptomic analysis of complex tissues^30,39,40^, and assessing both canonical and alternative AHR ligands^41^ have bolstered knowledge of the pleiotropic roles AHR signalling can play in vivo. Despite this, reagents that enable AHR activity to be reliably monitored in living tissues remain surprisingly limited. For example, although several models are available to examine the impact of deleting *Ahr* or *Ahr*-associated genes in cells, tissues and animals^42–44^, routine cellular tracking of AHR/AHR-associated proteins using conventional antibody-based flow cytometry has remained elusive. Instead, endogenous tagging of AHR and AHR-associated proteins with fluorophores or other molecular adapters has been used to visualise these proteins in experimental settings in vitro or ex vivo^45^.

Since *Cyp1a1* expression is dependent on AHR activation^13–15^, induction of *Cyp1a1* is a useful surrogate for AHR activity. On this basis, several prior studies generated transgenic mouse lines that contained *Cyp1a1* promoter sequences, derived from rat or human, cloned upstream of reporter genes such as *CAT, luciferase* or *GFP*^46–49^, (reviewed in^44^). Such reporters have provided invaluable tools for assessing *Cyp1a1* responses to different environmental stimulants, but may not contain the full repertoire of genetic regulatory elements available within the endogenous *Cyp1a1* locus, which normally serve to control expression in different cell types and developmental stages. To address this gap, and moreover, to develop robust murine reporters that enable endogenous AHR activity to be longitudinally and non-invasively imaged, we inserted firefly luciferase (*Fluc*) into the 3’UTR of the mouse *Cyp1a1* locus. Analogous approaches had previously been used by our group to successfully derive mouse embryonic stem cells (ESCs) and animal models in which the allelic expression of imprinted genes can be visualised throughout lifespan and across generations^50–52^ or that allow dystrophin and utrophin gene expression to be simultaneously imaged throughout mouse development^53^.

Here we describe the generation and properties of a bespoke *Cyp1a1-Fluc* knock-in mouse reporter that was designed to sensitively monitor AHR activity across murine life course. We show that *Cyp1a1* expression remains low during foetal development, but is inducible upon exposure to AHR ligands. In adults, in vivo challenge with the high affinity endogenous ligand 6-formylindolo[3,2-β]carbazole (FICZ), or the environmental pollutant and AHR agonist 3-methylcholanthrene (3-MC), results in strong *Cyp1a1*-derived bioluminescence signal in intestine, lung, liver and heart tissues. We show that dietary exposure to indole 3-carbinol (I3C) also provokes durable *Cyp1a1* expression within the gastrointestinal track and among discrete populations of cells resident in the adult lung.

## Results

### A luciferase-based endogenous *Cyp1a1* reporter that monitors AHR activity in vivo

**Fig. 1:**
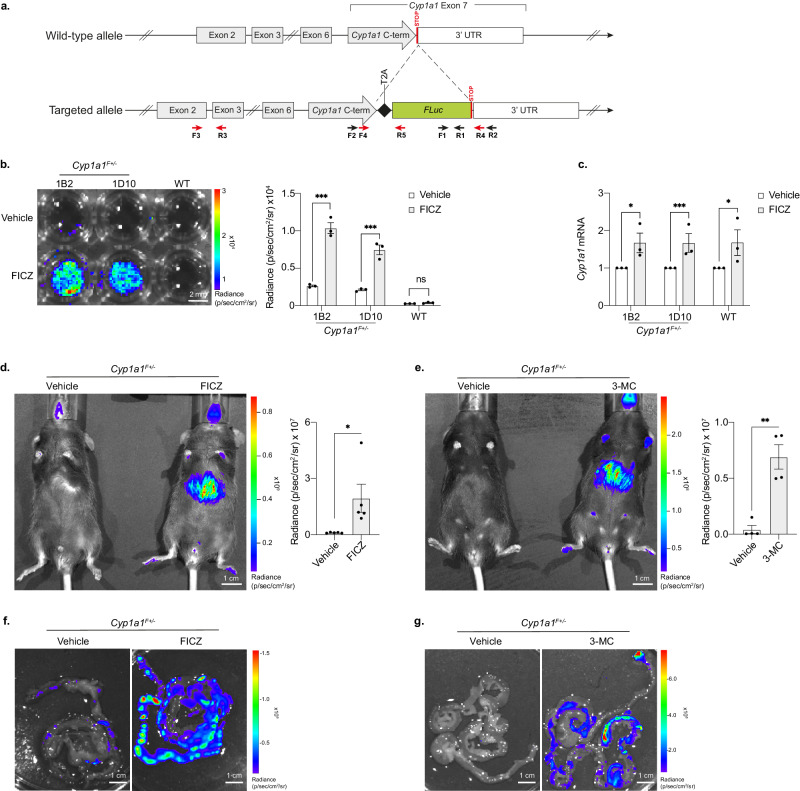
Generating a luciferase-based allelic reporter of endogenous *Cyp1a1* expression. **a** Diagram of the gene targeting strategy used to generate knock in *Cyp1a1*^*F*^ reporter mESCs and mouse lines. A firefly luciferase (*Fluc*) gene was inserted just before the stop codon in exon 7 of endogenous *Cyp1a1*, and it was separated from the C-terminal region by a T2A sequence. Arrows indicate PCR primers: F1 and R1 were used for firefly luciferase (*Fluc*) genotyping (see S1d), F2 and R2 for *Cyp1a1* wild type allele genotyping (see S1d), F3 and R3 for total *Cyp1a1* mRNA quantification, F4 and R4 for *Cyp1a1* wild type allele specific mRNA quantification and F4 and R5 for *Cyp1a1*^*F*^ knock in allele specific mRNA quantification (see S2c-d). Red arrows indicate those used for mRNA quantification while black arrows are for genotyping. **b** Representative bioluminescence image (left) and flux quantification (right) of two *Cyp1a1*^*F*^ mESC clones (1B2 and 1D10) shown alongside the parental wild type (WT) mESC line after 4 h exposure to FICZ or vehicle. Bars show the mean of 3 replicates +/- SEM, with paired *t*-tests to compare vehicle with FICZ treated samples for each cell line. **c** RT-qPCR of *Cyp1a1* mRNA expression from *Cyp1a1*^*F*^ (1B2 and 1D10) and WT mESCs following 4 h FICZ treatment. Levels of *Cyp1a1* mRNA are normalised to *Gapdh* mRNA and shown relative to the vehicle control. Bars show mean (*n* = 3) +/- SEM with paired *t*-tests to compare vehicle with FICZ treated samples. **d** Bioluminescence imaging of heterozygote (*Cyp1a1*^*F+/-*^) adult mice 5 h post intraperitoneal (IP) injection with vehicle or FICZ. Representative image (left) and whole-body quantification (right). Bars show mean (*n* = 5) +/- SEM with an unpaired *t*-test to compare vehicle with FICZ treated sample. **e** Representative image (left) and whole-body quantification (right) of *Cyp1a1*^*F+/-*^ adult mice 5 h post IP injection with vehicle or 3-MC. Bars show mean (*n* = 4) +/- SEM with an unpaired *t*-test to compare vehicle and 3-MC treated samples. **f**, **g** Representative bioluminescence images (*n* = 3) of intestine dissected from *Cyp1a1*^*F+/-*^ mice 5 h post FICZ (**f**) or 3-MC (**g**) injection. *p < 0.05, **p < 0.01, ***p < 0.001.

To generate a reporter for *Cyp1a1-*expression in pluripotent mouse ESCs, firefly luciferase (*Fluc*) was inserted into the 3’UTR of the endogenous *Cyp1a1* locus, downstream of exon 7. This targeting strategy was designed to be minimally disruptive since self-cleaving T2A sites ensure that Cyp1a1 and luciferase polypeptides are generated from a single *Cyp1a1-Fluc* mRNA transcript, while preserving the function of the targeted allele^54,55^ (Fig. 1a summarises the targeting strategy as well as primers used for genotyping (F1, R1, F2, R2) and mRNA expression (F3, R3, F4, R4, R5)). Using this approach, two heterozygous *Cyp1a1*^*F*+/-^ ESC clones were generated, 1B2 and 1D10, which were verified by DNA sequencing. Treatment of either clone with FICZ for 5 h resulted in significant bioluminescence signal (blue-green) upon addition of D-luciferin (Fig. 1b, and quantified in bar chart, right). Consistent with this we detected significant increases in *Cyp1a1* mRNA following FICZ exposure, as compared to vehicle treated controls (Fig. 1c). As anticipated, control wild type ESCs (WT) showed a similar increase in *Cyp1a1* expression in response to FICZ treatment (Fig. 1c), without detectable bioluminescence signal (Fig. 1b, WT). Exposure of 1B2, 1D10 clones and WT ESCs to 3-MC also provoked an increase in *Cyp1a1* mRNA detection relative to vehicle controls (Fig. S1a). These data are consistent with increased *Cyp1a1* expression and luciferase activity in targeted ESC clones following exposure to AHR ligands. Although Cyp1a1-luciferase activity was basal/ low in ESCs, this could be further reduced by treatment with the AHR inhibitor BAY-218^56^ (Fig. S1b). Pre-treatment of these cells with BAY-218 was insufficient however to fully block FICZ-induced *Cyp1a1* upregulation (Fig. S1c). Clone 1B2 was then used to create mouse lines where AHR-ligand responses could be investigated in a whole organism setting.

*Cyp1a1-Fluc* knock-in animals were derived and genotyped as described in Fig. S1d. Whole body bioluminescence imaging of these mice revealed *Cyp1a1*-derived flux signal in living (anaesthetised) heterozygous *Cyp1a1*^*F+/-*^ animals 5 h after injection with FIZC or 3-MC (Fig. 1d, e respectively, compare with *Cyp1a1*^*F+/-*^ animals injected with vehicle alone). Bioluminescence was detected in multiple tissues and verified posthumously, following dissection. For example, elevated bioluminescence signals throughout the gastrointestinal tracts of FICZ or 3-MC treated animals were detected (Fig. 1f, g, respectively), as compared to vehicle treated *Cyp1a1*^*F+/-*^ controls. These data show that exposure to AHR ligands in vivo induces the expression of *Cyp1a1*-derived luciferase in reporter mice, that can be visualised and quantified by bioluminescent imaging. In addition, bioluminescent imaging of embryoid bodies, that were derived from targeted ESCs in vitro, showed strong signal induction following exposure to FICZ (Fig. S1e). Taken together, these data show that the engineered *Cyp1a1-Fluc* (*Cyp1a1*^*F*^) models described here offer an opportunity to directly image AHR activity in vivo, ex vivo and within complex in vitro-derived cultures, such as embryoid bodies.

### Durable *Cyp1a1-Fluc* expression in vivo following challenge with FICZ or 3-MC

**Fig. 2:**
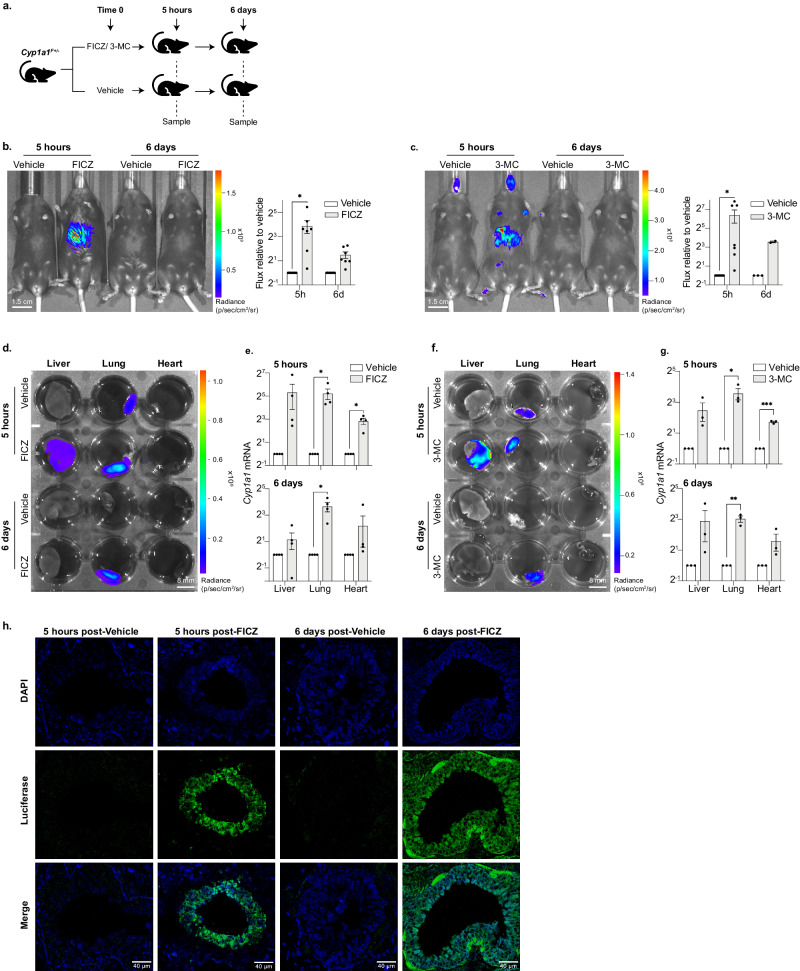
Longitudinal imaging of AHR activity following FICZ and 3-MC exposure in vivo reveals prolonged *Cyp1a1* expression in the lung. **a** Experimental design diagram outlining the exposure duration and sampling protocol. *Cyp1a1*^*F+/-*^ mice were intraperitoneal (IP) injected with either an AHR ligand (FICZ or 3-MC) or vehicle (corn oil). Mice were sampled for in vivo and ex vivo bioluminescence imaging, RT-qPCR or immunofluorescence analysis at 5 h and 6 days post ligand exposure. Diagram was generated using Adobe Illustrator and Microsoft PowerPoint ‘icons’. **b** In vivo bioluminescence imaging of *Cyp1a1*^*F+/-*^ adult mice 5 h and 6 days post FICZ injection. Representative image (left) and whole-body quantification of radiance (right). Bars show mean flux relative to vehicle treated control +/- SEM. Unpaired *t*-tests were used to compare vehicle with FICZ-treated mice for each time point (*p < 0.05). **c** As in (**b**) but following 3-MC IP injection. **d**, **e** Liver, lung, and heart were dissected from adult *Cyp1a1*^*F+/-*^ mice 5 h and 6 days after FICZ IP injection. **d** Representative bioluminescence image of tissues following 2 min incubation in D-luciferin. **e** RT-qPCR for *Cyp1a1* expression at 5 h (upper) and 6 days (lower) post FICZ injection. *Cyp1a1* mRNA is normalised against *18* *S* rRNA and *Tbp* mRNA and each sample is shown relative to its vehicle treated counterpart. Bars show mean +/- SEM and *t*-tests with Holm-Sidak multiple comparison testing were used to compare vehicle with FICZ treated samples (adjusted *p*-values are shown *p < 0.05, ***p < 0.001). **f**, **g** As in (**d**, **e**) for 3-MC treated samples. (**b**, **c**, **e**, **g**). Graphs are shown in Log2 scale. **h** Anti-luciferase immunofluorescence staining of 20 micron sections of lung tissue collected 5 h (first two panels) or 6 days (final two panels) post injection with FICZ or vehicle. Upper panel shows staining for nuclei alone (DAPI, blue), middle shows anti-luciferase staining (green) and lower panel shows the two stains merged.

To further investigate the duration of AHR-ligand responses in vivo, we performed longitudinal imaging and molecular analyses to track *Cyp1a1-Fluc* expression over time. Heterozygous reporter mice were examined 5 h and 6 days after FICZ or 3-MC challenge, as outlined in Fig. 2a. In response to FICZ, whole body bioluminescence imaging detected a variable but significant increase in flux signal relative to vehicle alone controls, that declined by day 6 (Fig. 2b shows representative images [left], and signal quantification [right]). In response to 3-MC, strong bioluminescent signal was evident at 5 h, that although reduced by day 6, remained above the levels seen in animals injected with vehicle alone (Fig. 2c). Close inspection of tissues dissected from animals sacrificed at each timepoint showed significant increases in bioluminescence and *Cyp1a1* mRNA expression in liver and lung tissues following FICZ exposure, as compared to vehicle alone controls (Fig. 2d, e) and showed that this persists in the lung for 6 days. Animals injected with 3-MC, a longer-lived AHR ligand^13,57,58^ showed broadly similar results (Fig. 2f, g). *Cyp1a1*-derived bioluminescent signal was also evident in brain, skin and thymus (Fig. S2a). Intraepithelial lymphocytes (IELs) that were isolated from the gut of 3-MC exposed animals were also bioluminescent (Fig. S2a, b). This pattern of expression is consistent with previous reports showing inducible AHR activity within these tissues^59–63^. Interestingly, although FICZ or 3-MC exposure provoked *Cyp1a1* upregulation in the heart (Fig. 2e and g), a corresponding signal was not immediately visible by bioluminescence (Fig. 2d and f), most likely because the heart is enriched with blood and absorbance can mask the detection of photons^53^.

Heterozygous (*Cyp1a1*^*F+/-*^) mice injected with either FICZ or 3-MC showed prominent and durable *Cyp1a1*-derived mRNA expression in lung tissues (Fig. 2e and g). To exclude that *Cyp1a1* expression had been inadvertently altered by *Fluc* insertion, we examined allelic expression arising from either the targeted (KI) or wild type (WT) locus. As shown in Fig. S2, we detected a similar increase in expression of *Cyp1a1* WT- and *Cyp1a1* KI-derived mRNA in lung tissue from mice exposed to FICZ (Fig. S2c), or to 3-MC (Fig. S2d). Induction of *Cyp1b1* and *Cyp1a1* were similar across a range of tissues (Fig. S2e), while *Cyp1a2*, a gene predicted to show a different pattern of expression^64^, was only transiently upregulated in the liver (Fig. S2f). Taken together, these results show that although FICZ and 3-MC provoke different kinetics of AHR activation and ligand clearance^13,57,58^, *Cyp1a1* responses to both agents in the lung were surprisingly long lived. Low-level expression of *Cyp1a1*-derived signal was noted in control (vehicle-treated) lung tissue (Fig. 2d and f, top row middle), a result that infers basal expression of *Cyp1a1* in adult mouse lung. To better identify cells within the lung that responded to AHR ligand, histology was performed using haematoxylin and eosin (H&E) (Fig. S2g) or DAPI (blue) and anti-luciferase to label cells expressing *Cyp1a1-Fluc* (green, Fig. 2h and S2h). We show that columnar epithelial cells surrounding bronchioles were intensely labelled with anti-luciferase antibody following exposure to FICZ (Fig. 2h), as well as some cells that lined smaller airways (Fig. S2h). Luciferase expression was also detected in other areas of the lung, including those associated with musculature (Fig. S2h). Remarkably, *Cyp1a1* signal was sustained in the lung 6 days after FICZ exposure and was prominent in cells that surrounded larger (>300 µm) bronchioles (Fig. 2h, right-hand panels).

### Expression of *Cyp1a1* during mouse ontogeny

**Fig. 3:**
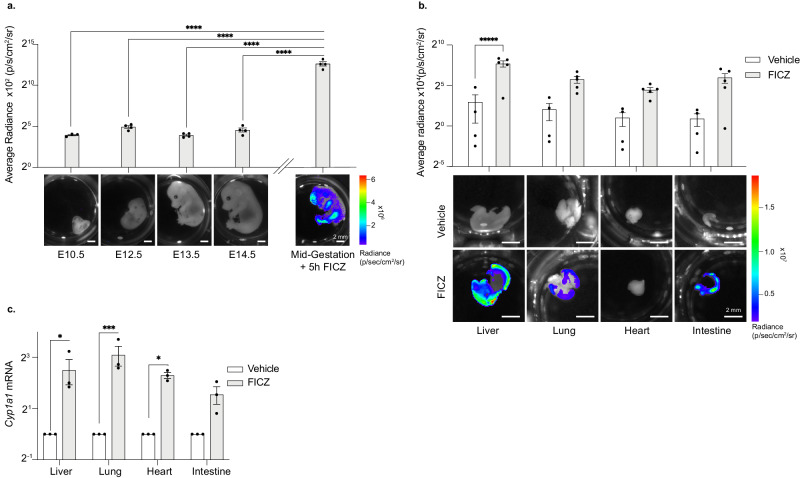
*Cyp1a1*^*F*^ reporter expression during ontogeny. **a** Bioluminescence images of *Cyp1a1*^*F+/-*^ mouse embryos collected at E10.5, E12.5, E13.5 and E14.5 stages of gestation (without AHR stimulation). A mid-gestation embryo which was incubated with FICZ for 5 h is shown alongside for comparison. Representative images are shown below and quantification above where bars show average radiance +/- SEM with a one-way ANOVA to compare the untreated embryos with the 20 nM FICZ treated embryo (*p* < 0.0001) with Dunnetts multiple comparison test. Adjusted *p*-values are shown ****p < 0.0001. **b** Liver, lung, heart and intestine were dissected from E14.5 *Cyp1a1*^*F+/-*^ embryos and incubated with vehicle or 20 nM FICZ for 5 h. Ex vivo bioluminescence images (upper) and quantification (lower) show increased luminescence in the liver, lung and intestine in response to FICZ. Bar graph shows average radiance +/- SEM with *t*-tests with Holm-Sidak multiple comparison testing were used to compare vehicle with FICZ treated samples (adjusted *p*-values are shown ****p < 0.0001). **c** RT-qPCR for *Cyp1a1* expression on samples from **b** *Cyp1a1* mRNA is normalised against *18* *S* rRNA and *Tbp* mRNA and each sample is shown relative to its vehicle treated counterpart. Bars show mean +/- SEM with Holm-Sidak multiple comparison testing were used to compare vehicle with FICZ treated samples (adjusted *p*-values are shown *p < 0.05, ***p < 0.001) to compare vehicle with FICZ treated samples, (*p < 0.05, ***p < 0.001). **a**–**c** Graphs are shown on a Log2 scale.

To explore when *Cyp1a1* is expressed during mouse development, we performed bioluminescence imaging and molecular analysis of embryos generated from mating *Cyp1a1-Fluc* heterozygote and wild type mice. In prior studies, using a transgenic mouse line containing 8.5 kb of the rat *Cyp1a1* promoter linked to *lac*Z, *Cyp1a1*-driven expression was reported in many tissues throughout stages E7-E14^65^. Despite this expectation, we did not observe any generalised expression of *Cyp1a1-*derived signal in *Cyp1a1*^*F*+/-^ reporter embryos sampled from E10 to E14.5 (Fig. 3a). We have previously shown that bioluminescence can be sensitively imaged in developing mouse embryos using a range of different luciferase reporter lines^50,51,53^, which excludes that failure to detect signal was simply due to a technical limitation in embryo imaging. Furthermore, exposure of E14.5 *Cyp1a1*^*F*+/-^ whole embryos or dissected tissues to FICZ ex vivo, resulted in abundant *Cyp1a1*-derived flux signal detection (Fig. 3a, b) and *Cyp1a1* mRNA expression in embryonic heart, lung, liver and intestine (Fig. 3c), as compared to vehicle controls. Taken together, these results clearly demonstrate that while *Cyp1a1* expression is normally low in the developing embryo, it can be induced upon exposure to AHR ligands. Differences between the results reported herein and those that were published previously^65^ might therefore reflect differences in pathogen load or commensal microbes resident within different mouse colonies.

### *Cyp1a1-Fluc* expression within the lung of reporter mice challenged with dietary I3C

**Fig. 4:**
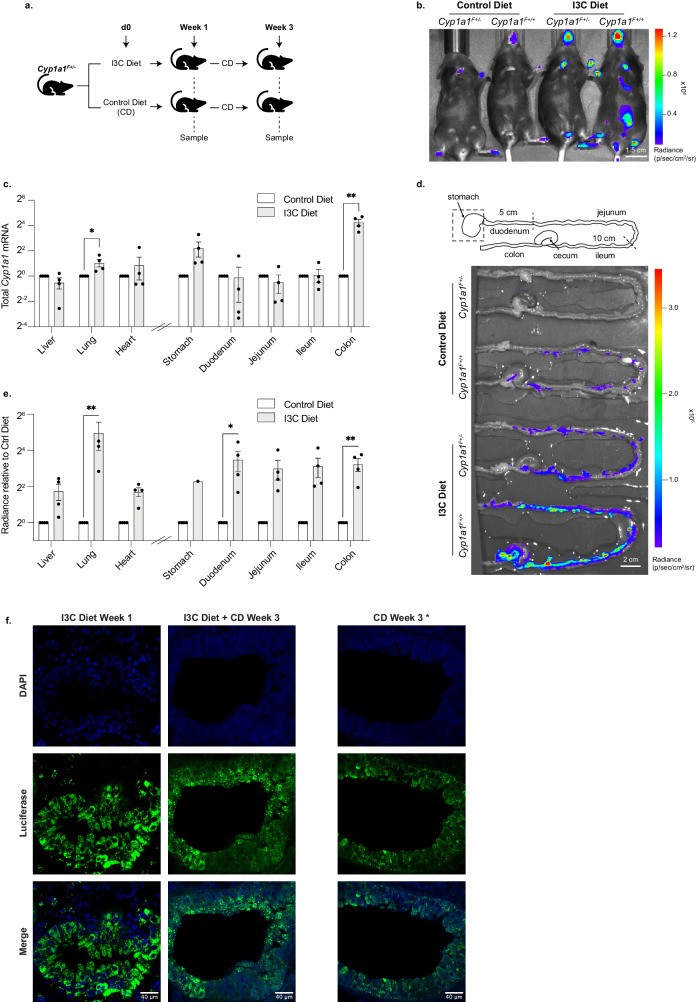
AHR activity in the intestine and lung of *Cyp1a1*^*F*^ reporter mice fed with purified diet supplemented with I3C. **a** Experimental design diagram outlining the exposure duration and sampling protocol. *Cyp1a1*^*F+/-*^ adult mice were fed either a purified control diet (CD), or the same diet supplemented with I3C for 1 week. At this point mice were either sampled or switched to CD for a further 2 weeks and sampled at the 3-week timepoint. Diagram was created in Adobe Illustrator using Microsoft PowerPoint, ‘icons’. **b** Representative bioluminescence image of *Cyp1a1*^*F+/-*^ and *Cyp1a1*^*F+/+*^ adult mice fed either purified control diet or I3C diet for 1 week. **c** RT-qPCR on tissues samples isolated from adult mice following 1 week of purified control diet or I3C diet. *Cyp1a1* mRNA was normalised against *18* *S* rRNA and *Tbp* mRNA and shown relative to the corresponding control diet sample. Unpaired *t*-tests were used to compare control diet with I3C diet (*p < 0.05, **p < 0.01). **d** Bioluminescence images of intestines dissected from *Cyp1a1*^*F+/-*^ and *Cyp1a1*^*F+/+*^ mice following 1 week of CD or I3C diet. Intestines are arranged corresponding to the gut diagram shown in above, with the stomach out of view of the image. Diagram generated by authors using Adobe Illustrator. **e** Quantification of bioluminescence imaging shown in (**d**) and S3a. *Cyp1a1*^*F+/-*^ radiance is shown relative to vehicle treated sample. Unpaired *t*-tests were used to compare control diet with I3C diet (*p < 0.05, **p < 0.01). **f** Anti-luciferase immunofluorescence staining of lung tissue following 1-week I3C diet (first panel); 1-week I3C diet followed by 2 weeks CD (middle panel); or 3 weeks CD (final panel). * Mice housed in a non-SPF environment. Upper panels show nuclei staining with DAPI (blue), middle panel shows luciferase (green) staining and lower panel shows both channels merged.

Whereas mice that have been raised in a conventional setting are known to display *Cyp1a1* expression, for example in intestinal epithelial cells and in associated immune cells, those raised in germ-free conditions or exposed to lower levels of microbial factors express *Ahr*, *Ahrr* and *Cyp1a1* at lower levels^66^. Exposure to I3C in diet, a natural product of glucobrassin hydrolysis, stimulates *Cyp1a1* activity in the intestine as well as in the liver^27,67^. Although I3C normally binds to AHR with low affinity, under acidic conditions I3C can be converted to indolo[3,2-β]carbazole, which has high affinity for AHR^67^. We examined the impact of dietary I3C on *Cyp1a1* expression using *Cyp1a1*^*F*+/-^ and *Cyp1a1*^*F*+/+^ reporter mice. Animals were fed purified diet with or without I3C and imaged after 1 week. To investigate the durability of I3C-induced *Cyp1a1* expression, mice that were exposed to control or I3C diet were then returned to normal chow for two further weeks before being imaged (Fig. 4a). As shown in Fig. 4b, *Cyp1a1*-derived bioluminescence signal was readily detected in heterozygous and homozygous animals that had been fed I3C diet, with prominent signal evident in dissected lung samples (Fig. S3a). Molecular analysis across of a range of different tissues confirmed elevated *Cyp1a1* mRNA expression in the lung and colon of I3C exposed animals, compared with control diet samples (Fig. 4c). Elevated bioluminescence signal was detected in the intestine of I3C-diet fed animals, as compared with control diet fed animals (Fig. 4d), and signal intensity was noticeably higher in homozygous (*Cyp1a1*^*F*+/+^) than heterozygous (*Cyp1a1*^*F*+/-^) samples. Bioluminescent imaging showed luciferase activity throughout the intestine of I3C-fed *Cyp1a1*^*F*+/-^ animals (Fig. 4d, quantified in e), however, standard molecular analyses of *Cyp1a1*-mRNA in isolated regions of the gut (Fig. 4c, right) detected significant increases in the colon, rather than more proximal regions. This most likely reflects a known limitation of standard ‘bulk’ RNA analysis, where gene expression is averaged across a population of different cell types, and then normalised to standard reference genes. In such a setting, rarer cells that express a gene of interest may be overlooked. In this context, IEL’s that were isolated directly from the gut (>95% CD45-positive) showed strong *Cyp1a1*-driven bioluminescence activity in response to IC3 (Figs. S3b and S3c), consistent with AHR activity being induced in gut-associated immune cells^59,62^.

To extend these findings and moreover to investigate *Cyp1a1* upregulation in the lung in response to dietary I3C, we performed immunofluorescence labelling using anti-luciferase antibody. We detected prominent labelling of columnar epithelium around lung bronchioles in mice fed I3C diet for a week (Fig. 4f, left). Two weeks later, after being returned to a normal diet, appreciable *Cyp1a1*-driven luciferase expression remained in lung tissues (Fig. 4f, centre). These data clearly showed that *Cyp1a1* expression by epithelial cells surrounding bronchioles were susceptible to dietary activation. We also noted that animals continuously fed purified control diet but housed in a non-SPF or conventional animal facility, also display elevated *Cyp1a1*-luciferase expression in such cells (Fig. 4f, right). Our results therefore suggest that prolonged AHR activation in the lung can be stimulated by multiple agents and encompass different routes of exposure. Taken together, these data show how *Cyp1a1- Fluc* reporter mice can be used to identify sites of prolonged AHR activity in vivo, for example within the mouse lung, and thereby illustrate their utility as dynamic sensors of environmentally induced AHR activation.

## Discussion

The aryl hydrocarbon receptor has fundamental roles in biology and AHR homologues are present in most animals from chordates to nematodes and molluscs^68–71^. Evidence from vertebrates and invertebrates suggest that AHR signalling is an ancestral process that has, for example, underwritten the parallel development of sensory neural systems in both phyla. In vertebrates, AHR plays a crucial role in mediating responses to xenobiotics and in modulating adaptive immune responses to metabolites generated through bacterial, dietary and environmental exposures. This is best illustrated in the mammalian gastrointestinal tract where constant exposure to microbes and dietary ligands requires an epithelial barrier equipped with immune surveillance to protect and maintain health^27,28,37,66^. While the importance of AHR is widely appreciated, investigating the impacts of dietary exposures and the mechanisms that can resolve or potentiate AHR activity in vivo remains a challenge. Towards this goal we produced a bespoke mouse reporter line in which AHR-induced expression of endogenous *Cyp1a1* could be visualised longitudinally in vivo using bioluminescence. We predicted that this could offer two major advantages. First, because bioluminescence imaging does not require external excitation to generate signal, unlike conventional fluorophore-based approaches that monitor gene activity^52^, we reasoned that this might improve signal detection by offering a high signal to noise ratio. Second, in contrast to previously generated *Cyp1a1*-promoter transgenic animals^46,48,49,65^, our strategy to create a ‘knock-in’ mouse by non-disruptive targeting of luciferase into the endogenous *Cyp1a1* locus should enable the normal dynamics of *Cyp1a1* expression to be accurately monitored. Our results show that *Cyp1a1*^*F+/-*^ mice respond appropriately to AHR ligands such as FICZ and 3-MC, or dietary exposure to I3C, by upregulating luciferase expression. Increased *Cyp1a1-Fluc* expression was detected by bioluminescence imaging and confirmed by measuring luciferase mRNA in tissues using quantitative RT-qPCR, and protein distribution by immunofluorescence labelling with luciferase-specific antibody.

*Cyp1a1-Fluc* adult mice housed in specific pathogen free conditions showed very low levels of luciferase reporter activity. Likewise, during foetal development we detected only minimal *Cyp1a1-Fluc* expression in embryos examined from E10.5 to E14.5. However, external exposure of mid-gestation embryos to FICZ (ex vivo) resulted in marked increases in *Cyp1a1-Fluc* expression, with bioluminescence signal evident in most major organs. These data support a view that AHR-signalling is inducible during mouse embryonic development^65^, as well as in differentiating ESCs^72^. Although prior studies with *Cyp1a1*-promoter transgenic mice have suggested that *Cyp1a1* expression is constitutive in embryos, with some evidence of temporal and spatial selectivity^65^, in our hands *Cyp1a1* expression was uniformly low (basal) throughout ontogeny but remained inducible. While such differences could conceivably indicate the presence of negative regulatory elements at the endogenous *Cyp1a1* locus, it is perhaps more likely that such discrepancies merely reflect differences in the maternal availability of AHR ligands in animals housed under different conditions. Interestingly, different inbred mouse strains are known to display different AHR ligand binding affinities^73^. The *Cyp1a1*^*F*^ reporter strain studied here is on a C57/Bl6 background and is therefore predicted to display high affinity AHR binding, by virtue of the *Ahrb2* allele. Future experiments will be required to assess detection in mouse strains that express low affinity AHR binding alleles (*Ahrd*), such as DBA2/SJ or CAST/Ei.

Inducible expression of *Cyp1a1* by alveolar and bronchiolar epithelial cells in response to smoking or hyperoxic conditions, has been described in humans and transgenic mice, respectively^74,75^. Here we show that *Cyp1a1* upregulation in bronchiolar epithelial cells was prolonged after exposure to either FICZ or to dietary I3C. The duration of *Cyp1a1* induction in these cells was longer than might be anticipated for ligands predicted to be susceptible to AHR-mediated metabolic degradation^27,76^. While the basis of this prolonged expression is not yet known, acute sensitivity of the respiratory system to altered AHR expression is well-documented^77^ as is the role of AHR in modulating inflammatory lung diseases such as asthma, COPD and silicosis (reviewed in^78^). AHR signalling is also heavily implicated in the development and progression of pulmonary arterial hypertension, with a recent study showing that lung endothelial cells, together with T cells and macrophages, are central for driving AHR-mediated pathology^79^. The provision of a bespoke *Cyp1a1* ‘knock-in’ reporter mouse line that accurately portrays the dynamics of AHR signalling longitudinally in vivo, will be of considerable value in evaluating the impacts and duration of repeated challenge in individual animals. Although in these studies we have used concentrations of AHR ligands that were defined by others^72,80–82^, our observation that *Cyp1a1* luminescence was induced in animals maintained on control diets but switched to a non-SPF facility, suggests that this model can detect endogenously-generated and physiologically relevant AHR activity. That said, it is also important to recognise that *ex vivo Cyp1a1* mRNA analysis and *Cyp1a1*-luciferase-based luminescence offer slightly different read-outs of AHR activity. This can be because of differences that stem from *Cyp1a1* mRNA detection and quantification (relative to ubiquitously expressed controls), mRNA stability, the translation and activity of luciferase proteins, and the sensitivity of photon detection (reviewed in^52^).

Monitoring AHR responses in vivo is particularly difficult in complex tissues such as lung. Recent single-cell transcriptomic analysis of developing mouse lung reveals a diverse mixture of cell types^83^, with eight different epithelial, six endothelial, and nine mesenchymal subtypes molecularly defined. Similar studies with human samples have confirmed this view, documenting a plethora of epithelial, endothelial, and mesenchymal cells that are integrated with immune cells to ensure airway development and function^84–86^. Using our *Cyp1a1-Fluc* reporters we have shown that exposure to FICZ or dietary I3C induces a prolonged AHR response where cells that line the bronchioles of the lung, as well as others, express luciferase and *Cyp1a1* mRNA. This aligns with single cell RNA-seq data generated as part of the mouse cell atlas project^87–89^ which showed *Cyp1a1* expression in different cell types in the lung including endothelial cells, myofibrogenic progenitors and smooth muscle. Our observation that dietary I3C exposure provokes prolonged activation of AHR in ‘barrier’ cell types in lung is important in understanding how encounter with respiratory pathogens may be affected by dietary or other environmental cues. It is well established that maternal diets enriched with AHR ligands can, for example, protect perinatal offspring from potentially lethal intestinal bacterial infection^90^ as well as ameliorate colitis in adult mice (reviewed in^1^). It is tempting to speculate that similar mechanisms operate in the lung where AHR signalling is known to afford anti-viral protection from agents such as Zika virus or SARS-CoV-2^91,92^, as well as mediating reduced lung capacity through overt inflammation and increased mucin production^93,94^.

Animal models that enable AHR activity to be quantitatively monitored throughout lifespan and in response to environmental changes including infection, diet and disease, offer important new tools for studying disease pathology and progression. The *Cyp1a1-Fluc* mice that we describe herein, will be useful for defining the cell types and precise mechanisms that underlie prolonged AHR activation in the lung. They also offer an unrivalled opportunity to discover new therapeutic interventions that can be used to alter the fine balance between ‘risk and benefit’ of modulating AHR activity in vivo, particularly with the aim of ameliorating disease processes.

## Methods

### Animal maintenance

All animal procedures were performed in accordance with the British Home Office Animal (Scientific Procedures) Act 1986. The mouse work was approved by the Imperial College AWERB committee and performed under a UK Home Office Project Licence and Personal Licences. Mice were housed in a SPF facility at temperatures of 21 + / − 2 °C; 45–65% humidity; 12 h light-dark cycle; with water and RM3 diet ad libitum. Tissues, wood blocks, and tunnels were used to enrich the environment. Experiments on adult mice were performed on animals between 3–16 weeks old.

### Generation of mESCs, mouse line and PCR genotyping

*Cyp1a1-Fluc* (referred to as *Cyp1a1*^*F*^) mESCs and mouse line were generated by OzGene, Australia. A firefly luciferase (*Fluc*) gene was inserted just before the stop codon in exon 7 of endogenous *Cyp1a1*, and it was separated from the C-terminal region by a T2A sequence (see Fig. 1a for details). Genotyping by PCR was carried out using HotStar Taq DNA Polymerase (Qiagen) according to manufacture conditions. Two independent PCR reactions were performed in parallel for each DNA sample. A first PCR reaction with two sets of primer pairs at a final concentration of 0.2 mM each: one specific for *Fluc* and another specific for a region of wild type *CD79b* (Chr11: 17714036-17714620) that serves as internal control. A second PCR reaction with only one pair of primers at a final concentration of 0.4 mM specific for the wild type allele of *Cyp1a1*. Primer sequences are indicated in Table 1.

**Table 1:** Primer sequences used in this study

Target	Orientation	Sequence (5’ – 3’)	Assay	Reference
*Fluc*	Forward (F1)	TTCCATCTTCCAGGGATACG	Genotyping	This study
*Fluc*	Reverse (R1)	ATCCAGATCCACAACCTTCG	Genotyping	This study
*Cyp1a1*	Forward (F2)	CTGTGAACACTTCCAAGTGC	Genotyping	This study
*Cyp1a1*	Reverse (R2)	TGTGCCCAGTGTGTGTTCAG	Genotyping	This study
*Cyp1a1*	Forward (F3)	CCTGTCCTCCGTTACCTGCC	RT-qPCR	This study
*Cyp1a1*	Reverse (R3)	AGGCTGTCTGTGATGTCCCG	RT-qPCR	This study
*Cyp1a1*	Forward (F4)	CCCGCTGTGAACACTTCCAA	Allele specific RT-qPCR	This study
*Cyp1a1*	Reverse (R4)	TGAATCACAGGAACAGCCACCT	Allele specific RT-qPCR	This study
*Fluc*	Reverse (R5)	AGTTGCTCTCCAGCGGTTCC	Allele specific RT-qPCR	This study
*CD79b*	Forward	GAGACTCTGGCTACTCATCC	Genotyping	This study
*CD79b*	Reverse	CCTTCAGCAAGAGCTGGGGAC	Genotyping	This study
*Gapdh*	Forward	AAGAGAGGCCCTATCCCAACTC	RT-qPCR	^95^
*Gapdh*	Reverse	TTGTGGGTGCAGCGAACTTTATTG	RT-qPCR	^95^
*Tbp*	Forward	GAAGAACAATCCAGACTAGCAGCA	RT-qPCR	^96^
*Tbp*	Reverse	CCTTATAGGGAACTTCACATCACAG	RT-qPCR	^96^
*18* *S* rRNA	Forward	GTAACCCGTTGAACCCCATT	RT-qPCR	^53^
*18* *S* rRNA	Reverse	CCATCCAATCGGTAGTAGCG	RT-qPCR	^53^
*Cyp1b1*	Forward	CCACCAGCCTTAGTGCAGAC	RT-qPCR	This study
*Cyp1b1*	Reverse	GGCCAGGACGGAGAAGAGT	RT-qPCR	This study
*Cyp1a2*	Forward	AGTACATCTCCTTAGCCCCAG	RT-qPCR	This study
*Cyp1a2*	Reverse	GGTCCGGGTGGATTCTTCAG	RT-qPCR	This study

### mESC culture and treatment

C57BL/6 knock-in mESCs clones and Bruce4 parental wild-type mESCs were cultured on a layer of mitotically-inactivated mouse embryonic fibroblasts on 0.1% gelatin-coated dishes with KnockOut Dulbecco’s Modified Eagle’s Medium (Gibco), supplemented with 15% fetal bovine serum (Gibco), 0.5% penicillin-streptomycin (Gibco), 0.1 mM non-essential amino acids (Gibco), 2 mM L-glutamine (Gibco), 0.1 mM 2-mercaptoetanol (Sigma), 10^3 ^U/mL of leukemia inhibitory factor (ESGRO, Millipore) and 2 mM of GSK-3 inhibitor IX (BIO, Selleckchem). Cells were incubated at 37 °C with 5% CO_2_ and split every 2–3 days. Cells were treated with 10 nM FICZ (BML-GR206-0100, Enzo), 1 nM 3-MC (213942, Sigma) or DMSO (Sigma) as vehicle control.

BAY-218 (S8842, Selleck Chemicals) was resuspended in DMSO. 1B2 *Cyp1a1*^*F*^ mESCs were treated with varying concentrations of BAY-218 for 24 h. For combined treatment, 20 nM FICZ or vehicle, was added to the culture medium 5 h prior to bioluminescence imaging.

Embryoid bodies were generated using the hanging drop method (Ohnuki and Kurosawa, 2013) and cultured in KnockOut Dulbecco’s Modified Eagle’s Medium (Gibco), supplemented with 20% fetal bovine serum (Gibco), 0.5% penicillin-streptomycin (Gibco), 0.1 mM non-essential amino acids (Gibco), 2 mM L-glutamine (Gibco) and 0.1 mM 2-mercaptoetanol (Sigma). 30 ml droplets containing around 1000 1B2 *Cyp1a1*^*F*^ mESCs were pipetted onto the lid of a petri dish and incubated at 37 °C with 5% CO_2_ for 3 days. The lower half of the dish was filled with PBS and the drops of mESCs were suspended over the PBS, hanging from the lid. The embryoid bodies were then washed from the lid into culture medium and grown for a further 2 or 4 days in suspension. Individual embryoid bodies were counted into 96 well plates at 1, 2, 4, 8 embryoid bodies per well and incubated with 20 nM FICZ or DMSO for 5 h.

### Animal studies

For in vivo experiments, adult mice were weighed and intraperitoneal (IP) injected with FICZ (SML1489, Sigma) or 3-MC (213942, Sigma) freshly prepared in warm corn oil (Sigma) at 10 mg/kg or 26.5 mg/kg, respectively. Corn oil was used as vehicle control.

For timed mating, an adult male was set up with 2 adult females and morning plug checking was performed. The females were separated from males upon observation of vaginal plugs, at which point they were considered to be at E0.5 developmental stage.

For diet studies, adult mice were fed with purified diet E157453-047 (D12450J) or E157453-047 (D12450J) containing 1000 mg/kg I3C (Sigma) provided by ssniff Spezialdiäten GmbH. Both diets were sterilized by gamma-irradiation at 25 kGy.

### Bioluminescence imaging

To image mESCs, D-luciferin (Perkin Elmer) was diluted in ESC medium to a final concentration of 150 μg/mL and added to mESCs 10 min prior to imaging. Cells were imaged using the IVIS Spectrum (Perkin Elmer) and Living Image software (version 4.3.1) to detect bioluminescence. All images were taken after 5 min exposure and at field of view (FOV) C with binning 4 and 0.5 depth using a stage temperature of 37 °C.

For in vivo bioluminescence imaging experiments, adult mice were intraperitoneal (IP) injected with 0.15 mg/g D-luciferin (Perkin Elmer), dissolved in dH_2_O. Mice were left conscious for 3 min to allow the D-luciferin to circulate systemically and then anesthetized through isoflurane inhalation. At 10 min post-injection, mice were imaged using the IVIS Spectrum. Adult mice were imaged for 3 min using FOV C or D, binning 1 and 1.5 depth using a stage temperature of 37 °C.

For ex vivo experiments, dissected tissues were incubated in 150 μg/mL D-luciferin in DMEM medium without Phenol Red (Gibco) for 2 min prior to imaging for 3 min at FOV C or D with binning 1 and 0.5 depth using a stage temperature of 37 °C.

For embryos imaging, pregnant females were first IP injected with 0.15 mg/g D-luciferin and left conscious for 3 min to allow the D-luciferin to circulate systemically, then mice were culled followed by embryo dissection. Embryos were placed in 24-well plates and incubated with freshly prepared 150 μg/mL D-luciferin in DMEM/F12 medium (Gibco) for 2 min prior to imaging on the IVIS for 1 min using FOV A, binning 4 and 0.75 depth.

Image analysis and bioluminescence quantification were carried out using the Living Image software (version 4.5.2) (Perkin Elmer). Briefly, regions of interest (ROI) were drawn around plate wells containing cells, tissues and embryos or around whole animals to calculate flux (p/s) and average radiance (p/s/cm^2^/sr) within the region.

### RNA extraction and RT-qPCR

RNA from mESC and tissue samples was purified using the RNeasy Mini Kit (Qiagen). Cells were lysed immediately after imaging with RLT buffer. Tissues were dissected and frozen in liquid nitrogen. Prior to RNA purification, frozen tissues were lysed in RLT buffer on the TissueLyser II (Qiagen) using 5 mm stainless steel beads (Qiagen) for 4 min at 24,000 rpm. Heart samples were incubated with 10 μg/ml Proteinase K at 55 °C for 1 h. All tissue samples were then centrifuged at top speed for 3 min and total RNA was purified from the supernatant using the RNeasy Mini Kit (Qiagen) according to the manufacturer’s instructions, including on-column DNase digestion step using an RNase-Free DNase Set (Qiagen). After quantification, 2 μg of total RNA was used to perform cDNA synthesis with 10 μM random primers using the SuperScript III Reverse Transcriptase Kit (Invitrogen), following manufacturer’s instructions.

RT-qPCR was performed with 0.4 μM primers and using the QuantiTect SYBR Green PCR mix (Qiagen). For allele specific amplification, a forward primer binding to exon 7 of *Cyp1a1* (F4) was combined either with a reverse primer binding to exon 7 of *Cyp1a1* (R4) to specifically amplify the *Cyp1a1* WT allele or with a reverse primer binding to the *Fluc* sequence (R5) to specifically amplify the *Cyp1a1-Fluc* allele. Positions of the primers are indicated in Fig. 1a. Primer sequences are indicated in Table 1. Samples were analysed in 3 technical replicates. PCR reactions were carried out in a CFX thermocycler (Bio-Rad) for 40 cycles of a 2-step amplification protocol consisting of 94 °C for 15 s and 60 °C for 30 s. A disassociation final step to calculate melting temperature was included in all RT-qPCR experiments.

### Immunofluorescent microscopy on frozen tissue sections

Mouse lung or colon tissue was dissected, fixed in 10% Formalin solution (Sigma Aldrich), incubated with 30% sucrose solution for 3 days at 4 °C and frozen in Optimal Cutting Temperature (OCT, Thermo) to form blocks. The tissue blocks were cryosectioned (20 µm) and mounted on microscope slides (Superfrost Plus Adhesion Microscope slides, VWR) and stored at -80 °C. Thawed sections were fixed in 2% Paraformaldehyde (Fluka) for 20 min at room temperature, rinsed with PBS and permeabilized with 0.4% Triton X-100 in PBS for 5 min at room temperature in Coplin jars. The tissue sections were blocked using 10% fetal calf serum in PBS for 20 min at room temperature inside a humidified chamber. The tissue sections were then incubated with anti-firefly luciferase (Abcam 185924) diluted 1:100 in Blocking Buffer overnight at 4 °C in a humidified chamber. The tissue sections were washed 3 × 5 min in Wash Buffer (PBS containing 0.2% BSA, 0.05% Tween 20) and then incubated with Alexa Flour-488 conjugated secondary antibody (Invitrogen 1874771) diluted 1:400 in Blocking Buffer for 1 h at room temperature in a humidified chamber. Following 2 × 5 min washes in Wash Buffer and 1 × 5 min wash in PBS, the sections were mounted in Vectorshield anti-fade mounting medium containing DAPI (Vector Laboratories). The tissue sections were imaged on a Leica Stellaris 5 confocal microscope using 63x objective, or Leica DM4B microscope using the 10x objective using LAS-X software. Image analysis was carried out using FiJI (version 2.14.0).

### Haematoxylin and Eosin staining

Surgically dissected lung tissue was fixed in 10% neutral buffered formalin solution for 24 h and transferred to 70% Ethanol prior to processing using Sakura Tissue-Tek VIP® 6 automated tissue processor. Briefly, lung tissues in embedding cassettes were dehydrated by progressing through steps of 70% ethanol for 45 min at 37 °C, 80% ethanol for 45 min at 37 °C, 90% ethanol for 30 min at 37 °C, 96% ethanol for 45 min at 37 °C, 100% ethanol for 30 min at 37 °C, 100% ethanol for 1 h at 37 °C, 100% ethanol for 1 h at 37 °C. Dehydrated samples are then cleared by three washes in Xylene for 30 min, 45 min and 1 h at 37 °C. Finally, specimens are infiltrated by two immersions in 62 °C paraffin wax for 45 min and 1 h, followed by two immersions in 62 °C paraffin wax for 30 min. The tissues were then embedded in paraffin-block using (Leica EG1160 Embedding Center) and 4 μm sections made using ThermoFisher scientific Microtome Microm HM355S and attached to slides.

Prior to staining, sections were deparaffinised by washing slides 3X in HistoclearTM for 2 min each, followed by three 2 min washes of 100% ethanol, before a final 2 min wash in dH_2_O. Slides were incubated for 60 s in Modified Mayer’s Haematoxylin (Lillie’s Modification) (DAKO), washed for 5 min in tap water and immersed for 2 s in Eosin followed by washing in dH_2_O. Prior to mounting coverslips with DPX mounting medium (Sigma) slides were dehydrated by three washes 100% ethanol for 2 min each and three washes in Histoclear for 2 min each.

The tissue sections were imaged using a Leica DM6000 microscope (10x objective) with a DFC 450 C4 colour camera and Leica LAS-X software.

### Isolation of intraepithelial lymphocytes (IELs) from small intestine

Small intestine was dissected, opened longitudinally, washed with 1X PBS and cut into small pieces before incubation with 100 mM DTT in RPMI/ 10% FBS for 20 min at room temperature. After centrifugation at 1500 rpm for 5 min, the pellet was resuspended in 10 ml of RPMI/10% FBS and vortexed at maximum speed for 4 min. Samples were filtered using 70 micron cell strainer to harvest lymphocyte-containing supernatant. Remaining intestinal pieces were resuspended in 10 ml of RPMI/10% FBS, vortexed again at maximum speed for 4 min and filtered through the same cell strainer to combine both fractions. The cell pellet was collected by centrifugation at 1500 rpm for 10 min. Lymphocytes were purified using an 80%: 40%: 20% Percoll gradient by centrifugation at 1800 rpm for 30 min at room temperature, with slow acceleration/deceleration settings. After gradient centrifugation, lymphocytes were collected from the 40% Percoll fraction and washed with 1X PBS at 1800 rpms for 5 min. To assess the purity of the collected samples, lymphocyte-containing cell suspensions were stained with anti-CD45-PerCP-Cy5.5 antibody (clone 30-F11, Cat No. 103112, Biolegend, dilution 1:100) for 1 h at 4 °C. After washing with 1X PBS, samples were stained with DAPI (Cat No. 130-111-570, Miltenyi) to assess cell viability and then analysed by flow cytometry in the BD FACS Aria III system. IEL preparations were routinely verified as being 90-95% CD45 positive. Supplementary Fig. 4 indicates an example of the gating strategy used.

### Statistics and reproducibility

Microsoft Excel was used for calculations with raw data and GraphPad Prism (version 8) was used for graph generation and statistical analysis. Graphs show the mean of experimental replicates and standard error (SEM), with specific details provided in the figure legends. All data points were taken from distinct samples and not repeatedly measured. Multi-group comparisons were tested using one-way ANOVAs with Dunnett’s or Sidak’s correction for multiple comparisons. Pair-wise comparisons were tested using a paired *t*-test with Holm-Sidak multiple comparison testing when multiple comparisons were made. Details are described in the figure legends.

All numerical source data is provided in Supplementary Data 1.

### Reporting summary

Further information on research design is available in the Nature Portfolio Reporting Summary linked to this article.

### Supplementary information

Supplementary Information

Description of Additional Supplementary Files

Supplementary Data 1

Reporting Summary

## Data Availability

All numerical source data is provided in Supplementary Data file 1.

## References

[1] Rothhammer V, Quintana FJ (2019). The aryl hydrocarbon receptor: an environmental sensor integrating immune responses in health and disease. Nat. Rev. Immunol..

[2] Esser C, Rannug A (2015). The aryl hydrocarbon receptor in barrier organ physiology, immunology, and toxicology. Pharmacol. Rev..

[3] Burbach KM, Poland A, Bradfield CA (1992). Cloning of the Ah-receptor cDNA reveals a distinctive ligand-activated transcription factor. Proc Natl Acad Sci USA.

[4] Ema M (1992). cDNA cloning and structure of mouse putative Ah receptor. Biochem. Biophys. Res. Commun..

[5] Nebert DW (2017). Aryl hydrocarbon receptor (AHR): “pioneer member” of the basic-helix/loop/helix per-Arnt-sim (bHLH/PAS) family of “sensors” of foreign and endogenous signals. Prog. Lipid Res..

[6] Bersten DC, Sullivan AE, Peet DJ, Whitelaw ML (2013). bHLH-PAS proteins in cancer. Nat. Rev. Cancer.

[7] Gutierrez-Vazquez C, Quintana FJ (2018). Regulation of the immune response by the aryl hydrocarbon receptor. Immunity.

[8] Shinde R, McGaha TL (2018). The aryl hydrocarbon receptor: connecting immunity to the microenvironment. Trends Immunol..

[9] Murray IA, Perdew GH (2020). How Ah receptor ligand specificity became important in understanding its physiological function. Int. J. Mol. Sci..

[10] Agus A, Planchais J, Sokol H (2018). Gut microbiota regulation of tryptophan metabolism in health and disease. Cell Host Microbe.

[11] Chiaro CR, Patel RD, Marcus CB, Perdew GH (2007). Evidence for an aryl hydrocarbon receptor-mediated cytochrome p450 autoregulatory pathway. Mol. Pharmacol..

[12] Rifkind AB (2006). CYP1A in TCDD toxicity and in physiology-with particular reference to CYP dependent arachidonic acid metabolism and other endogenous substrates. Drug Metab. Rev..

[13] Wei YD, Bergander L, Rannug U, Rannug A (2000). Regulation of CYP1A1 transcription via the metabolism of the tryptophan-derived 6-formylindolo[3,2-b]carbazole. Arch Biochem. Biophys..

[14] Wei YD, Helleberg H, Rannug U, Rannug A (1998). Rapid and transient induction of CYP1A1 gene expression in human cells by the tryptophan photoproduct 6-formylindolo[3,2-b]carbazole. Chem. Biol. Interact.

[15] Heath-Pagliuso S (1998). Activation of the Ah receptor by tryptophan and tryptophan metabolites. Biochemistry.

[16] Mescher M, Haarmann-Stemmann T (2018). Modulation of CYP1A1 metabolism: from adverse health effects to chemoprevention and therapeutic options. Pharmacol. Ther..

[17] Mimura J, Ema M, Sogawa K, Fujii-Kuriyama Y (1999). Identification of a novel mechanism of regulation of Ah (dioxin) receptor function. Genes Dev..

[18] MacPherson L (2014). Aryl hydrocarbon receptor repressor and TiPARP (ARTD14) use similar, but also distinct mechanisms to repress aryl hydrocarbon receptor signaling. Int. J. Mol. Sci..

[19] Stockinger B, Shah K, Wincent E (2021). AHR in the intestinal microenvironment: safeguarding barrier function. Nat. Rev. Gastroenterol. Hepatol..

[20] Ma W (2022). Kynurenine produced by tryptophan 2,3-dioxygenase metabolism promotes glioma progression through an aryl hydrocarbon receptor-dependent signaling pathway. Cell Biol. Int..

[21] Xiong J (2020). Aryl hydrocarbon receptor mediates Jak2/STAT3 signaling for non-small cell lung cancer stem cell maintenance. Exp. Cell Res..

[22] Pan ZY (2018). Activation and overexpression of the aryl hydrocarbon receptor contribute to cutaneous squamous cell carcinomas: an immunohistochemical study. Diagn Pathol..

[23] Mohamed HT (2019). Inflammatory breast cancer: activation of the aryl hydrocarbon receptor and its target CYP1B1 correlates closely with Wnt5a/b-beta-catenin signalling, the stem cell phenotype and disease progression. J. Adv. Res..

[24] Mian C (2014). AHR over-expression in papillary thyroid carcinoma: clinical and molecular assessments in a series of Italian acromegalic patients with a long-term follow-up. PLoS One.

[25] To KK, Yu L, Liu S, Fu J, Cho CH (2012). Constitutive AhR activation leads to concomitant ABCG2-mediated multidrug resistance in cisplatin-resistant esophageal carcinoma cells. Mol. Carcinog..

[26] Su JM, Lin P, Wang CK, Chang H (2009). Overexpression of cytochrome P450 1B1 in advanced non-small cell lung cancer: a potential therapeutic target. Anticancer Res..

[27] Schiering C (2017). Feedback control of AHR signalling regulates intestinal immunity. Nature.

[28] Metidji A (2018). The environmental sensor AHR protects from inflammatory damage by maintaining intestinal stem cell homeostasis and barrier integrity. Immunity.

[29] Murray IA, Patterson AD, Perdew GH (2014). Aryl hydrocarbon receptor ligands in cancer: friend and foe. Nat. Rev. Cancer.

[30] Panda SK (2023). Repression of the aryl-hydrocarbon receptor prevents oxidative stress and ferroptosis of intestinal intraepithelial lymphocytes. Immunity.

[31] Ly M (2019). Diminished AHR signaling drives human acute myeloid leukemia stem cell maintenance. Cancer Res..

[32] Singh KP (2014). Loss of aryl hydrocarbon receptor promotes gene changes associated with premature hematopoietic stem cell exhaustion and development of a myeloproliferative disorder in aging mice. Stem Cells Dev..

[33] Singh KP, Wyman A, Casado FL, Garrett RW, Gasiewicz TA (2009). Treatment of mice with the Ah receptor agonist and human carcinogen dioxin results in altered numbers and function of hematopoietic stem cells. Carcinogenesis.

[34] Opitz CA (2011). An endogenous tumour-promoting ligand of the human aryl hydrocarbon receptor. Nature.

[35] Rothhammer V (2016). Type I interferons and microbial metabolites of tryptophan modulate astrocyte activity and central nervous system inflammation via the aryl hydrocarbon receptor. Nat. Med..

[36] Shinde R (2018). Apoptotic cell-induced AhR activity is required for immunological tolerance and suppression of systemic lupus erythematosus in mice and humans. Nat. Immunol..

[37] Rosser EC (2020). Microbiota-derived metabolites suppress arthritis by amplifying Aryl-hydrocarbon receptor activation in regulatory B cells. Cell Metab..

[38] Granados JC (2022). AHR is a master regulator of diverse pathways in endogenous metabolism. Sci. Rep..

[39] Cannon AS (2022). AhR activation leads to attenuation of murine autoimmune hepatitis: single-cell RNA-Seq analysis reveals unique immune cell phenotypes and gene expression changes in the liver. Front. Immunol..

[40] Dean JW (2023). The aryl hydrocarbon receptor cell intrinsically promotes resident memory CD8(+) T cell differentiation and function. Cell Rep..

[41] Stinn A, Furkert J, Kaufmann SHE, Moura-Alves P, Kolbe M (2021). Novel method for quantifying AhR-ligand binding affinities using microscale thermophoresis. Biosensors (Basel).

[42] Finn RN (2007). The physiology and toxicology of salmonid eggs and larvae in relation to water quality criteria. Aquat Toxicol..

[43] Noda S (2003). Gene expression of detoxifying enzymes in AhR and Nrf2 compound null mutant mouse. Biochem. Biophys. Res. Commun..

[44] Hannon SL, Ding X (2022). Assessing cytochrome P450 function using genetically engineered mouse models. Adv. Pharmacol.

[45] Degrelle SA, Ferecatu I, Fournier T (2022). Novel fluorescent and secreted transcriptional reporters for quantifying activity of the xenobiotic sensor aryl hydrocarbon receptor (AHR). Environ. Int..

[46] Jones SN, Jones PG, Ibarguen H, Caskey CT, Craigen WJ (1991). Induction of the Cyp1a-1 dioxin-responsive enhancer in transgenic mice. Nucleic Acids Res..

[47] Campbell SJ, Carlotti F, Hall PA, Clark AJ, Wolf CR (1996). Regulation of the CYP1A1 promoter in transgenic mice: an exquisitely sensitive on-off system for cell specific gene regulation. J. Cell Sci.

[48] Galijatovic A (2004). The human CYP1A1 gene is regulated in a developmental and tissue-specific fashion in transgenic mice. J. Biol. Chem..

[49] Operana TN, Nguyen N, Chen S, Beaton D, Tukey RH (2007). Human CYP1A1GFP expression in transgenic mice serves as a biomarker for environmental toxicant exposure. Toxicol Sci..

[50] Van de Pette M (2017). Visualizing changes in Cdkn1c expression links early-life adversity to imprint Mis-regulation in adults. Cell Rep..

[51] Van de Pette M (2022). Epigenetic changes induced by in utero dietary challenge result in phenotypic variability in successive generations of mice. Nat. Commun..

[52] Dimond A, Van de Pette M, Fisher AG (2020). Illuminating epigenetics and inheritance in the immune system with bioluminescence. Trends Immunol..

[53] Gleneadie HJ (2023). Endogenous bioluminescent reporters reveal a sustained increase in utrophin gene expression upon EZH2 and ERK1/2 inhibition. Commun Biol..

[54] Gao SY, Jack MM, O’Neill C (2012). Towards optimising the production of and expression from polycistronic vectors in embryonic stem cells. PLoS One.

[55] Toutounchian JJ, McCarty JH (2017). Selective expression of eGFP in mouse perivascular astrocytes by modification of the Mlc1 gene using T2A-based ribosome skipping. Genesis.

[56] Gutcher I (2019). Blocking tumor-associated immune suppression with BAY-218, a novel, selective aryl hydrocarbon receptor (AhR) inhibitor. Cancer Res..

[57] Riddick DS, Huang Y, Harper PA, Okey AB (1994). 2,3,7,8-Tetrachlorodibenzo-p-dioxin versus 3-methylcholanthrene: comparative studies of Ah receptor binding, transformation, and induction of CYP1A1. J. Biol. Chem..

[58] Bergander L (2004). Metabolic fate of the Ah receptor ligand 6-formylindolo[3,2-b]carbazole. Chem. Biol. Interact.

[59] Li Y (2011). Exogenous stimuli maintain intraepithelial lymphocytes via aryl hydrocarbon receptor activation. Cell.

[60] Kyoreva M (2021). CYP1A1 enzymatic activity influences skin inflammation Via regulation of the AHR pathway. J. Invest Dermatol..

[61] Kimura E, Tohyama C (2017). Embryonic and postnatal expression of aryl hydrocarbon receptor mRNA in mouse brain. Front. Neuroanat..

[62] Stockinger B, Di Meglio P, Gialitakis M, Duarte JH (2014). The aryl hydrocarbon receptor: multitasking in the immune system. Annu. Rev. Immunol..

[63] Ma N, He T, Johnston LJ, Ma X (2020). Host-microbiome interactions: the aryl hydrocarbon receptor as a critical node in tryptophan metabolites to brain signaling. Gut Microbes.

[64] Dey A, Jones JE, Nebert DW (1999). Tissue- and cell type-specific expression of cytochrome P450 1A1 and cytochrome P450 1A2 mRNA in the mouse localized in situ hybridization. Biochem. Pharmacol..

[65] Campbell SJ (2005). The murine Cyp1a1 gene is expressed in a restricted spatial and temporal pattern during embryonic development. J. Biol. Chem..

[66] Obata Y (2020). Neuronal programming by microbiota regulates intestinal physiology. Nature.

[67] Bjeldanes LF, Kim JY, Grose KR, Bartholomew JC, Bradfield CA (1991). Aromatic hydrocarbon responsiveness-receptor agonists generated from indole-3-carbinol in vitro and in vivo: comparisons with 2,3,7,8-tetrachlorodibenzo-p-dioxin. Proc. Natl Acad. Sci. USA.

[68] Hahn ME (2002). Aryl hydrocarbon receptors: diversity and evolution. Chem. Biol. Interact.

[69] Hahn ME (2006). Unexpected diversity of aryl hydrocarbon receptors in non-mammalian vertebrates: insights from comparative genomics. J. Exp. Zool A Comp. Exp. Biol..

[70] Hahn ME, Karchner SI, Merson RR (2017). Diversity as opportunity: insights from 600 million years of AHR evolution. Curr. Opin. Toxicol..

[71] Mulero-Navarro S, Fernandez-Salguero PM (2016). New trends in Aryl hydrocarbon receptor biology. Front. Cell Dev. Biol..

[72] Gialitakis M (2017). Activation of the Aryl hydrocarbon receptor interferes with early embryonic development. Stem Cell Rep..

[73] Thomas RS, Penn SG, Holden K, Bradfield CA, Rank DR (2002). Sequence variation and phylogenetic history of the mouse Ahr gene. Pharmacogenetics.

[74] Jiang W, Couroucli XI, Wang L, Barrios R, Moorthy B (2011). Augmented oxygen-mediated transcriptional activation of cytochrome P450 (CYP)1A expression and increased susceptibilities to hyperoxic lung injury in transgenic mice carrying the human CYP1A1 or mouse 1A2 promoter in vivo. Biochem. Biophys. Res. Commun..

[75] Saarikoski ST (1998). Localization of CYP1A1 mRNA in human lung by in situ hybridization: comparison with immunohistochemical findings. Int. J. Cancer.

[76] Rannug A (2020). How the AHR became important in intestinal homeostasis-A diurnal FICZ/AHR/CYP1A1 feedback controls both immunity and immunopathology. Int. J. Mol. Sci..

[77] Chiba T (2011). Arylhydrocarbon receptor (AhR) activation in airway epithelial cells induces MUC5AC via reactive oxygen species (ROS) production. Pulm Pharmacol. Ther..

[78] Beamer CA, Shepherd DM (2013). Role of the aryl hydrocarbon receptor (AhR) in lung inflammation. Semin. Immunopathol..

[79] Masaki T (2021). Aryl hydrocarbon receptor is essential for the pathogenesis of pulmonary arterial hypertension. Proc. Natl Acad. Sci. USA.

[80] Wiggins BG (2023). Endothelial sensing of AHR ligands regulates intestinal homeostasis. Nature.

[81] Ehrlich AK, Pennington JM, Bisson WH, Kolluri SK, Kerkvliet NI (2018). TCDD, FICZ, and other high affinity AhR ligands dose-dependently determine the fate of CD4+ T cell differentiation. Toxicol. Sci..

[82] Major J (2023). Endothelial AHR activity prevents lung barrier disruption in viral infection. Nature.

[83] Negretti NM (2021). A single-cell atlas of mouse lung development. Development.

[84] Schupp JC (2021). Integrated single-cell atlas of endothelial cells of the human lung. Circulation.

[85] Madissoon E (2023). A spatially resolved atlas of the human lung characterizes a gland-associated immune niche. Nat. Genet..

[86] He P (2022). A human fetal lung cell atlas uncovers proximal-distal gradients of differentiation and key regulators of epithelial fates. Cell.

[87] Han X (2018). Mapping the mouse cell atlas by microwell-seq. Cell.

[88] Fei L (2022). Systematic identification of cell-fate regulatory programs using a single-cell atlas of mouse development. Nat. Genet..

[89] Wang R (2023). Construction of a cross-species cell landscape at single-cell level. Nucleic Acids Res..

[90] Lu P (2021). Maternal aryl hydrocarbon receptor activation protects newborns against necrotizing enterocolitis. Nat. Commun..

[91] Giovannoni F (2020). AHR is a Zika virus host factor and a candidate target for antiviral therapy. Nat. Neurosci..

[92] Giovannoni F (2021). AHR signaling is induced by infection with coronaviruses. Nat. Commun..

[93] Liu Y (2020). Mucus production stimulated by IFN-AhR signaling triggers hypoxia of COVID-19. Cell Res..

[94] Giovannoni F, Quintana FJ (2021). SARS-CoV-2-induced lung pathology: AHR as a candidate therapeutic target. Cell Res..

[95] Veland N (2019). DNMT3L facilitates DNA methylation partly by maintaining DNMT3A stability in mouse embryonic stem cells. Nucleic Acids Res..

[96] Veazey KJ, Golding MC (2011). Selection of stable reference genes for quantitative rt-PCR comparisons of mouse embryonic and extra-embryonic stem cells. PLoS One.

