# Supplementary material for: Bioluminescence imaging of Cyp1a1-luciferase reporter mice demonstrates prolonged activation of the aryl hydrocarbon receptor in the lung
Source: Commun Biol. 2024 Apr 10;7:442. doi: 10.1038/s42003-024-06089-6 (PMC11006662; doi:10.1038/s42003-024-06089-6)
Supplement: Supplementary file 1 — Supplementary Information [file 42003_2024_6089_MOESM1_ESM.pdf]

Supplementary Figures

Bioluminescence imaging of *Cyp1a1*-luciferase reporter mice demonstrates prolonged activation of the aryl hydrocarbon receptor in the lung

Nicolas Veland[#1], Hannah J Gleneadie[#1], Karen E Brown[1], Alessandro Sardini[2], Joaquim Pombo[3], Andrew Dimond[1,8], Vanessa Burns[1], Karen Sarkisyan[4], Chris Schiering[5], Zoe Webster[6], Matthias Merkenschlager[7] & Amanda G Fisher[1,8*]

1. Epigenetic Memory Group, MRC Laboratory of Medical Sciences, Imperial College London Hammersmith Hospital Campus, Du Cane Road, London, W12 OHS, UK.
2. Whole Animal Physiology and Imaging, MRC Laboratory of Medical Sciences, Imperial College London, Hammersmith Hospital Campus, Du Cane Road, London, W12 0HS, UK.
3. Senescence Group, MRC Laboratory of Medical Sciences, Imperial College London Hammersmith Hospital Campus, Du Cane Road, London, W12 0HS, UK.
4. Synthetic Biology Group, MRC Laboratory of Medical Sciences, Imperial College London Hammersmith Hospital Campus, Du Cane Road, London, W12 0HS, UK.
5. Inflammation and Obesity Group, MRC Laboratory of Medical Sciences, Imperial College London Hammersmith Hospital Campus, Du Cane Road, London, W12 0HS, UK.
6. Transgenics & Embryonic Stem Cell Facility, MRC Laboratory of Medical Sciences, Imperial College London Hammersmith Hospital Campus, Du Cane Road, London, W12 0HS, UK.
7. Lymphocyte Development Group, MRC Laboratory of Medical Sciences, Imperial College London Hammersmith Hospital Campus, Du Cane Road, London, W12 0HS, UK.
8. Department of Biochemistry, University of Oxford, Oxford OX1 3QU, United Kingdom

*e-mail correspondence; amanda.fisher@bioch.ox.ac.uk
[#] These authors contributed equally

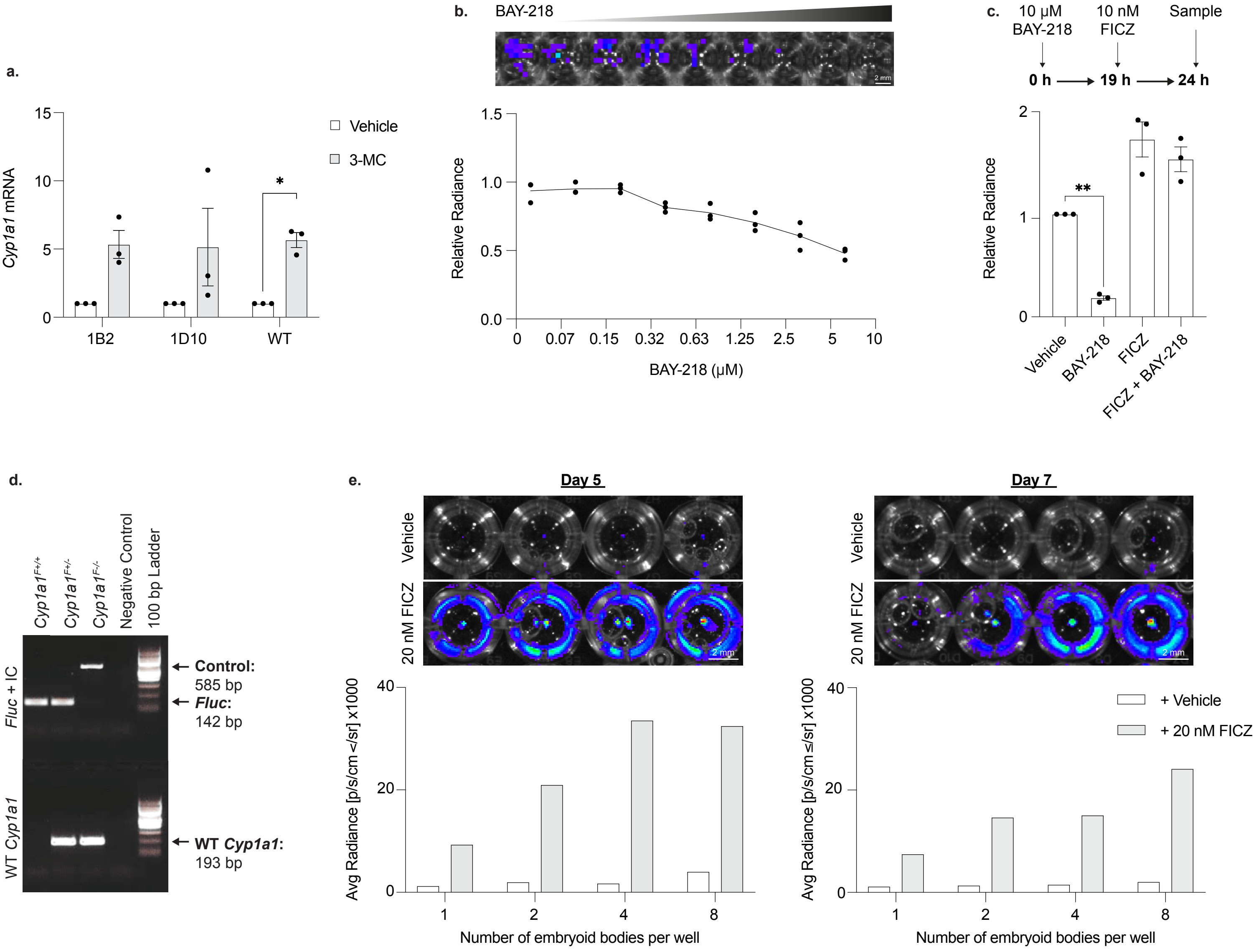
a.
Cyp1a1 mRNA
15
10
5
0
1B2
1D10
WT
Vehicle
3-MC
*
b.
BAY-218
2 mm
Relative Radiance
1.5
1.0
0.5
0.0
0
0.07
0.15
0.32
0.63
1.25
2.5
5
10
BAY-218 (μM)
c.
10 μM
BAY-218
10 nM
FICZ
Sample
0 h
19 h
24 h
Relative Radiance
2
1
0
**
Vehicle
BAY-218
FICZ
FICZ + BAY-218
d.
$Cyp1a1^{F+/+}$
$Cyp1a1^{F+/-}$
$Cyp1a1^{F-/-}$
Negative Control
100 bp Ladder
Fluc + IC
WT Cyp1a1
Control:
585 bp
Fluc:
142 bp
WT Cyp1a1:
193 bp
e.
Day 5
Day 7
Vehicle
20 nM FICZ
2 mm
Avg Radiance [p/s/cm²/sr] x1000
40
20
0
1
2
4
8
Number of embryoid bodies per well
+ Vehicle
+ 20 nM FICZ

Supplementary Figure 1:

a. RT-qPCR of *Cyp1a1* mRNA expression from two *Cyp1a1*^{F} mESC clones (1B2 and 1D10) and WT mESCs following 4-hour 3-MC treatment. Levels of *Cyp1a1* mRNA are normalised to *Gapdh* mRNA and shown relative to the vehicle control. Bars show mean (n=3) +/- SEM with paired t-tests to compare vehicle with 3-MC treated samples. **b-c.** Bioluminescence imaging of 1B2 *Cyp1a1*^{F} mESCs following treatment with AHR inhibitor BAY-218. **b**. 1B2 mESCs were treated with an increasing dose of BAY-218 for 24 hours. Graph shows mean (n=3) quantification of bioluminescence signal relative to vehicle only control, with representative image of bioluminescent wells shown above. **c.** 1B2 cells were treated with 10 mM BAY-218 for 24 hours with 10 nM FICZ, or vehicle added for the final 5 hours of treatment. Bars show mean (n=3) radiance relative to vehicle only control +/- SEM with one -way ANOVA with Dunnetts correction for multiple comparisons. Schematic of the treatment strategy is shown above. **d.** Representative image of an agarose gel with PCR results for genotyping of *Cyp1a1*^{F} mice. Two independent PCR reactions were performed in parallel for each DNA sample. The upper section of the gel illustrates a PCR reaction with two sets of primer pairs: one specific for the *Fluc* gene which amplifies a PCR product of 142 bp and another specific for a region of wild-type *CD79b* (Chr11: 17714036-17714620) that serves as internal control (IC) and amplifies a PCR product of 585 bp. The lower section of the gel illustrates a PCR reaction with only one pair of primers specific for the wild type allele of *Cyp1a1* (WT *Cyp1a1*) and amplifies a PCR product of 193 bp. Arrows indicate the respective sizes of PCR products amplified in the reactions. Examples of homozygous (*Cyp1a1*$^{F+/+}$) and heterozygous (*Cyp1a1*$^{F+/-}$) DNA samples are shown. DNA from the parental Bruce 4 mESC line was used as wild type (*Cyp1a1*$^{F-/-}$) control and the negative control was a non-template PCR reaction. Primers for *Fluc* are labelled as F1 and R1 while primers for WT *Cyp1a1* are labelled as F2 and R2, and their respective sequence locations are indicated in Figure 1a. **e.** Representative bioluminescence imaging of embryoid bodies (EBs) generated from 1B2 *Cyp1a1*^{F} mESCs using the hanging drop method in which ESCs were grown as droplets without LIF for 3 days and cell clusters were grown in suspension for a further 2 (Day 5, left) or 4 (Day 7, right) days. Individual embryoid bodies were counted into a 96 well plate at 1, 2, 4 or 8 EBs per well and treated with 20 nM FICZ or vehicle for 5 hours. Graph shows average radiance per well (n=1) with representative images of the bioluminescence signal shown above.

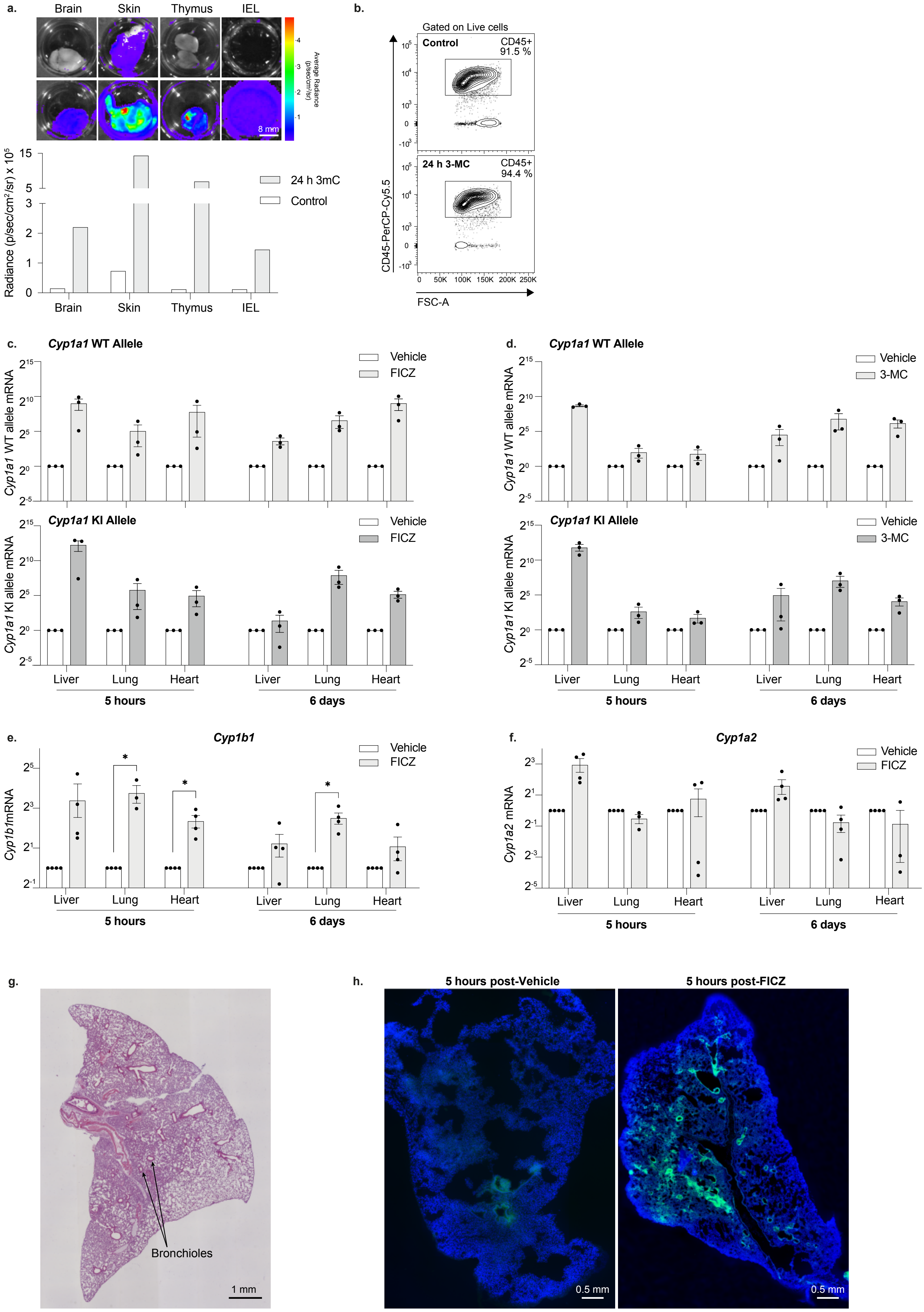

a.
Brain
Skin
Thymus
IEL
Average Radiance (p/sec/cm²/sr)
8 mm
Radiance (p/sec/cm²/sr) x 10⁵
24 h 3mC
Control
b.
Gated on Live cells
Control
CD45+ 91.5 %
24 h 3-MC
CD45+ 94.4 %
CD45-PerCP-Cy5.5
FSC-A
c.
Cyp1a1 WT Allele
Vehicle
FICZ
Cyp1a1 WT allele mRNA
Cyp1a1 KI Allele
Cyp1a1 KI allele mRNA
d.
3-MC
e.
Cyp1b1
Cyp1b1mRNA
f.
Cyp1a2
Cyp1a2 mRNA
Liver
Lung
Heart
5 hours
6 days
g.
Bronchioles
1 mm
h.
5 hours post-Vehicle
5 hours post-FICZ
0.5 mm

Supplementary Figure 2: Expression of AHR target genes in the lung following FICZ exposure.

a. Representative bioluminescence imaging of dissected tissues (brain, skin and thymus) and intraepithelial lymphocytes (IELs) isolated from the gut of *Cyp1a1*$^{F+/+}$ mice 24 hours following 3-MC IP injection. Graph shows radiance (n=1) with a representative image of the bioluminescent tissues shown above. **b.** Flow cytometry analysis indicating percentage of CD45+ cells in the gut IELs cell preparations shown in **a**. **c-d.** Allele specific RT-qPCR of tissues dissected from adult *Cyp1a1*$^{F+/-}$ mice 5 hours or 6 days post FICZ (**c**), 3-MC (**d**) or vehicle IP injection. Locations of primers which specifically amplify the WT *Cyp1a1* allele (F4 and R4) or knock in *Cyp1a1*^{F} allele (F4 and R5) are indicated in Figure 1a. **e-f.** Tissues were dissected from adult mice 5 hours and 6 days post FICZ or vehicle IP injection. RT-qPCR for *Cyp1b1* (**e**) and *Cyp1a2* (**f**). **c-f.** Levels of mRNA were normalised to *18S* rRNA and *Tbp* mRNA and results are shown relative to the corresponding vehicle treated sample. Bars show mean +/- SEM with t-tests with Holm-Sidak multiple comparison testing to compare vehicle with FICZ treated tissues, adjusted p values are shown (*$p<0.05$). **g**. Haematoxylin & eosin (H&E) staining of wax embedded *Cyp1a1*$^{F+/-}$ lung sections, with examples of bronchioles highlighted with arrows. **h.** Anti-luciferase immunofluorescence staining of lung tissue dissected from mice 5 hours after FICZ or vehicle IP injection. Representative images of whole lung sections, DAPI shown in blue and anti-luciferase shown in green.

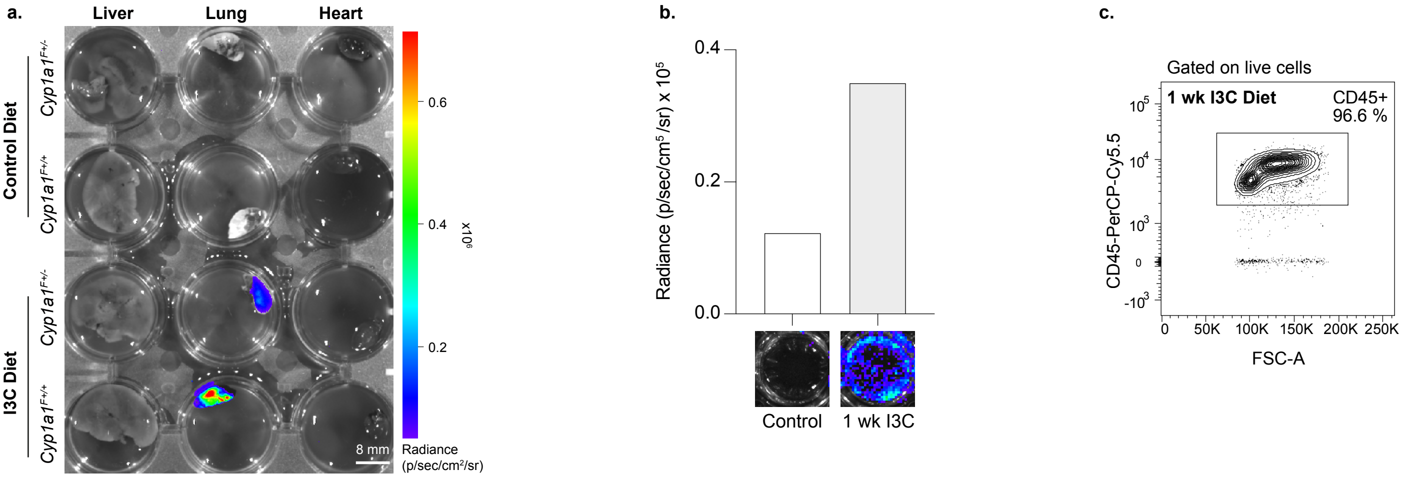

a.
Liver
Lung
Heart
Control Diet
I3C Diet
Cyp1a1F+/-
Cyp1a1F+/+
Cyp1a1F+/-
Cyp1a1F+/+
0.6
0.4
0.2
x10⁶
8 mm
Radiance (p/sec/cm²/sr)
b.
Radiance (p/sec/cm⁵/sr) x 10⁵
0.4
0.2
0.0
Control
1 wk I3C
c.
Gated on live cells
1 wk I3C Diet
CD45+
96.6 %
CD45-PerCP-Cy5.5
FSC-A
0
50K
100K
150K
200K
250K

Supplementary Figure 3: AHR activity in the lung of *Cyp1a1*^{F} reporter mice following I3C diet. Corresponding to Figure 4.

a. *Cyp1a1*$^{F+/-}$ and *Cyp1a1*$^{F+/+}$ adult mice were fed purified control diet or I3C diet for one week following which liver, lung and heart were dissected and analysed by *ex vivo* bioluminescence imaging. **b.** Bioluminescence imaging of intraepithelial lymphocytes (IELs) isolated from the gut of *Cyp1a1*$^{F+/+}$ mice following one week with I3C diet. Graph shows radiance (n=1) with a representative image of the bioluminescent cells shown below. **c.** Flow cytometry analysis indicating the percentage of CD45+ cells in the gut IELs cell preparation shown in **b.**

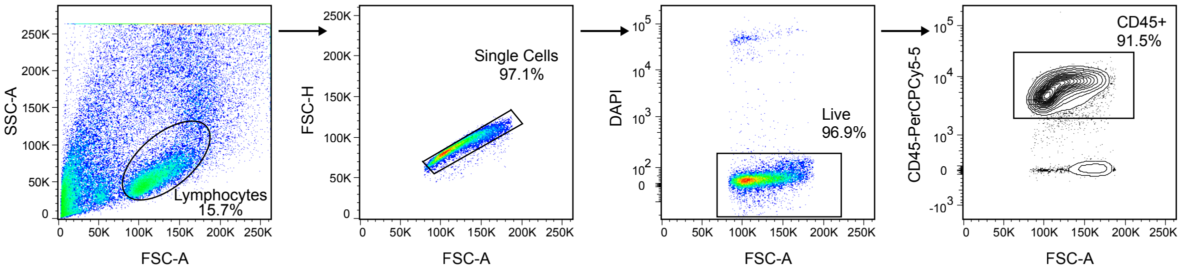
SSC-A
250K
200K
150K
100K
50K
0
Lymphocytes
15.7%
0
50K
100K
150K
200K
250K
FSC-A
FSC-H
Single Cells
97.1%
FSC-A
DAPI
Live
96.9%
FSC-A
CD45-PerCPCy5-5
CD45+
91.5%
FSC-A

Supplementary Figure 4: Gating strategy used for flow cytometry analysis.

Representative images of the gating strategy for flow cytometry analysis on purified intraepithelial lymphocytes from small intestine of adult control mouse (vehicle treated). Arrows indicate direction of gating from the selected population and percentage values are relative to parental population. Antibody for CD45 was used as marker to identify immune cells.
