# Supplementary material for: Bioluminescence imaging of Cyp1a1-luciferase reporter mice demonstrates prolonged activation of the aryl hydrocarbon receptor in the lung
Source: Commun Biol. 2024 Apr 10;7:442. doi: 10.1038/s42003-024-06089-6 (PMC11006662; doi:10.1038/s42003-024-06089-6)
Supplement: Supplementary file 2 — Description of Additional Supplementary Files [file 42003_2024_6089_MOESM2_ESM.pdf]

Description of Additional Supplementary Files

File name: Supplementary Data 1

Description: Numerical source data corresponding to all graphs within the figures and supplementary figures.
