# Supplementary material for: Bioluminescence imaging of Cyp1a1-luciferase reporter mice demonstrates prolonged activation of the aryl hydrocarbon receptor in the lung
Source: Commun Biol. 2024 Apr 10;7:442. doi: 10.1038/s42003-024-06089-6 (PMC11006662; doi:10.1038/s42003-024-06089-6)
Supplement: Supplementary file 3 — Supplementary Data 1 [file 42003_2024_6089_MOESM3_ESM.pdf]

1b

	Radiance x 1000					
	Vehicle			FICZ		
1B2	2.84	2.45	2.6	9.95	9.35	11.8
1D10	1.88	2.17	2.26	6.2	7.92	8.26
Wild type	0.28	0.256	0.276	0.282	0.382	0.457

1c

	Relative Expression					
	Vehicle			FICZ		
1B2	1	1	1	1.489	1.342	2.187
1D10	1	1	1	1.38	1.453	2.166
Wild type	1	1	1	1.181	1.522	2.334

1d

Radiance x 10^7	
Vehicle	FICZ
0.0826	0.873
0.0869	1.2
0.133	4.9
0.12	1.15
0.103	1.58

1e

Radiance x 10^7	
Vehicle	3-MC
0.00682	0.875
0.00495	0.497
0.00387	0.508
0.15	0.882

S1a

	Relative Expression					
	Vehicle			3-MC		
1B2	1	1	1	7.35216464	4.65240087	4.02045091
1D10	1	1	1	10.7907254	1.62109573	3.03431859
Wild type	1	1	1	6.11329995	4.57751869	6.28988676

S1b

218 Concentr	Relative Radiance		
0	1	1	1
0.078125	0.849	0.98	0.9808
0.15625	0.928	1	0.9254
0.3125	0.922	0.98	0.9552
0.625	0.816	0.78	0.8491
1.25	0.754	0.73	0.8439
2.5	0.646	0.69	0.7773
5	0.502	0.61	0.7043
10	0.43	0.51	0.4986

S1c

Relative Expression			
Vehicle	BAY-218	FICZ	ICZ + BAY-218
1	0.142	1.39	1.32
1	0.221	1.92	1.55
1	0.187	1.88	1.74

S1e

D5

nber of Ebs/\	+ Vehicle	+ 20 nM FICZ
**1**	1.29	9.37
**2**	2.01	21
**4**	1.78	33.6
**8**	4.06	32.5

D7

nber of Ebs/\	+ Vehicle	+ 20 nM FICZ
**1**	1.18	7.52
**2**	1.38	14.7
**4**	1.58	15.1
**8**	2.1	24.2

2b

	Flux relative to vehicle													
	Ctrl							FICZ						
**5h**	1	1	1	1	1	1	1	10.9	12.2	43.2	3.54	12.3	19.1	1.27
**6d**	1	1	1	1	1	1	1	1.95	1.95	4.78	2.15	4.46	3.09	1.21

2c

	Flux relative to vehicle													
	Ctrl							FICZ						
**5h**	1	1	1	1	1	1	1	168	171	230	7.52	4.68	1.44	9.26
**6d**	1	1	1					11.1	12.6	0.734				

2d

	mRNA relative to vehicle															
	5h Ctrl				5h FICZ				6d Ctrl				6d FICZ			
**Liver**	1	1	1	1	32.5362	114.069	7.68052	5.40734	1	1	1	1	2.13968	1.80478	0.29261	4.6984
**Lung**	1	1	1	1	69.5616	19.7302	37.9017	22.1854	1	1	1	1	19.6898	11.077	5.12707	14.3641
**Heart**	1	1	1	1	7.44658	7.92588	3.45463	9.80453	1	1	1	1	1.52309	1.19921	1.74092	13.872

2e

	mRNA relative to vehicle											
	5h Ctrl			5h 3mC			6d Ctrl			6d 3mC		
**Liver**	1	1	1	9.91837	2.822138	4.02135	1	1	1	16.222	4.100025	1.82521
**Lung**	1	1	1	8.28483	17.69184	9.64876	1	1	1	9.83336	5.965905	8.64153
**Heart**	1	1	1	3.44236	3.06217	3.39958	1	1	1	2.18329	5.204992	1.64709

S2a

	adiance relative to vehicl	
	Control	3-MC
**Brain**	0.154	2.21
**Skin**	0.74	14.4
**Thymus**	0.127	7.02
**IEL**	0.123	1.46

S2b

WT only		mRNA relative to vehicle					
		Ctrl			FICZ		
**5 h**	**Liver**	1	1	1	960.773	34.1543	580.026
	**Lung**	1	1	1	87.2338	3.09422	10.9856
	**Heart**	1	1	1	632.821	5.95819	30.2827
**6 d**	**Liver**	1	1	1	10.4883	20.1861	6.72187
	**Lung**	1	1	1	203.995	51.5752	34.6176

	Heart	1	1	1	1047.51	461.066	92.1234

		mRNA relative to vehicle					
**KI only**		Ctrl			FICZ		
**5 h**	Liver	1	1	1	7682.47	170.214	7040.24
	Lung	1	1	1	154.504	3.63742	12.2718
	Heart	1	1	1	73.6814	4.94657	16.6449
**6 d**	Liver	1	1	1	6.2784	0.18978	1.55158
	Lung	1	1	1	536.001	120.716	72.926
	Heart	1	1	1	60.6196	30.7096	18.9983

**S2c**		mRNA relative to vehicle					
**WT only**		Ctrl			3-MC		
**5 h**	Liver	1	1	1	394.673	357.989	489.307
	Lung	1	1	1	2.88735	1.68262	7.75453
	Heart	1	1	1	2.36459	1.30622	6.81158
**6 d**	Liver	1	1	1	53.8135	14.925	1.70632
	Lung	1	1	1	40.9659	263.422	34.014
	Heart	1	1	1	113.079	85.8199	19.8545

		mRNA relative to vehicle					
**KI only**		Ctrl			3-MC		
**5 h**	Liver	1	1	1	5688.94	3563	1675.6
	Lung	1	1	1	4.16727	2.12097	12.753
	Heart	1	1	1	1.95291	2.02535	5.99943
**6 d**	Liver	1	1	1	90.9207	3.75351	1.11977
	Lung	1	1	1	88.0072	271.278	50.5736
	Heart	1	1	1	18.8229	27.7062	5.3943

**S2e**		mRNA relative to vehicle							
Cyp1b1		Ctrl				FICZ			
**5 h**	Liver	1	1	1	1	26.3807	2.83621	9.1709	3.33024
	Lung	1	1	1	1		11.4285	21.3242	7.85773
	Heart	1	1	1	1	7.67424	2.73893	3.96918	6.06613
**6 d**	Liver	1	1	1	1	1.97197	0.57378	2.04023	4.78369
	Lung	1	1	1	1	3.27556	5.04655	8.56658	5.83172
	Heart	1	1	1	1	0.84996	1.34201	1.74233	4.53241

**S2f**		mRNA relative to vehicle							
Cyp1a2		Ctrl				FICZ			
**5 h**	Liver	1	1	1	1	10.8784	12.4052	3.48365	4.29222
	Lung	1	1	1	1		0.42606	0.90812	0.76285
	Heart	1	1	1	1	0.05517	3.4818	3.16773	0.09842
**6 d**	Liver	1	1	1	1	5.74859	1.72174	1.62782	2.95903
	Lung	1	1	1	1	0.1116	1.16348	0.67457	0.43463
	Heart	1	1	1	1	0.13376		0.06401	1.46446

3a

Average radiance x 10^2				
**E10.5**	**E12.5**	**E13.5**	**E14.5**	**Mid-Gestation + 5h FICZ**
16.2	29.2	17.6	22.5	6120
13.4	22	17.5	23.6	9300
16.9	38	12.5	14.9	6180
	34.1	13.8	34.7	3860

**3b**	Average radiance x 10^4									
	+ DMSO					+ FICZ				
**Liver**	27.2	0.434	0.179	3.34		189	230	10.7	323	289
**Lung**	11.4	0.429	0.261	4.66		27.3	68.3	16	104	54.3
**Heart**	4.38	0.133	0.264	3.39		18.7	23.5	8.45	36.4	23.4
**Intestine**	3.96	0.522	0.102	2.96		26.9	47.6	0.809	131	108

**3c**	mRNA relative to vehicle					
	+ DMSO			+ FICZ		
**Liver**	1	1	1	3.5969	4.0141	9.4278
**Lung**	1	1	1	13.056	6.6355	6.0119
**Heart**	1	1	1	5.635	4.257	4.8964
**Intestine**	1	1	1	1.758	2.9145	4.1506

4c

	mRNA relative to vehicle							
	Control Diet				I3C Diet			
**Liver**	1	1	1	1	0.66	0.81	1.16	0.17
**Lung**	1	1	1	1	1.51	3.16	1.3	2.32
**Heart**	1	1	1	1	4.8	0.65	1.16	0.64
**Stomach**	1	1	1	1	9.79	2.05	2.15	4.59
**Duodenum**	1	1	1	1	0.49	3.01	0.1	0.14
**Jejunum**	1	1	1	1	1.69	0.26	0.24	0.7
**Ileum**	1	1	1	1	2.11	0.97	0.48	0.72
**Colon**	1	1	1	1	25.9	23.3	15.73	11.08

**4e**	Flux relative to vehicle							
	Control Diet				I3C Diet			
**Liver**	1	1	1	1	4.77062147	2.0429207	1.23255814	5.43457627
**Lung**	1	1	1	1	77.1374587	7.15533259	22.8361742	18.7348106
**Heart**	1	1	1	1	3.50662589	1.73461854	4.35253296	3.46934307
**Stomach**	1	1	1	1				4.89178773
**Duodenum**	1	1	1	1	3.21096654	6.30691898	13.3505345	22.1125577
**Jejunum**	1	1	1	1	6.98726738	3.41062802	5.29560976	16.2790698
**Ileum**	1	1	1	1	4.05672515	4.56946984	8.8686768	17.5592034
**Colon**	1	1	1	1	12.2830031	3.86915888	13.594533	7.7883651

S3b

Average Radiance x10^4	
**Control**	**7d I3C**
0.123	0.35
