# Supplementary material for: Bioluminescence imaging of Cyp1a1-luciferase reporter mice demonstrates prolonged activation of the aryl hydrocarbon receptor in the lung
Source: Commun Biol. 2024 Apr 10;7:442. doi: 10.1038/s42003-024-06089-6 (PMC11006662; doi:10.1038/s42003-024-06089-6)
Supplement: Supplementary file 4 — Reporting Summary [file 42003_2024_6089_MOESM4_ESM.pdf]

nature portfolio

Corresponding author(s): Amanda G. Fisher

Last updated by author(s): Mar 13, 2024

Reporting Summary

Nature Portfolio wishes to improve the reproducibility of the work that we publish. This form provides structure for consistency and transparency in reporting. For further information on Nature Portfolio policies, see our Editorial Policies and the Editorial Policy Checklist.

Statistics

For all statistical analyses, confirm that the following items are present in the figure legend, table legend, main text, or Methods section.

n/a	Confirmed	
☐	☒	The exact sample size (*n*) for each experimental group/condition, given as a discrete number and unit of measurement
☐	☒	A statement on whether measurements were taken from distinct samples or whether the same sample was measured repeatedly
☐	☒	The statistical test(s) used AND whether they are one- or two-sided<br>*Only common tests should be described solely by name; describe more complex techniques in the Methods section.*
☐	☒	A description of all covariates tested
☐	☒	A description of any assumptions or corrections, such as tests of normality and adjustment for multiple comparisons
☐	☒	A full description of the statistical parameters including central tendency (e.g. means) or other basic estimates (e.g. regression coefficient) AND variation (e.g. standard deviation) or associated estimates of uncertainty (e.g. confidence intervals)
☐	☒	For null hypothesis testing, the test statistic (e.g. *F*, *t*, *r*) with confidence intervals, effect sizes, degrees of freedom and *P* value noted<br>*Give P values as exact values whenever suitable.*
☒	☐	For Bayesian analysis, information on the choice of priors and Markov chain Monte Carlo settings
☒	☐	For hierarchical and complex designs, identification of the appropriate level for tests and full reporting of outcomes
☒	☐	Estimates of effect sizes (e.g. Cohen's *d*, Pearson's *r*), indicating how they were calculated

Our web collection on statistics for biologists contains articles on many of the points above.

Software and code

Policy information about availability of computer code

Data collection	• IVIS images were generated using an IVIS Spectrum (Perkin Elmer) and the Living Image software (version 4.3.1).<br>• The Bio-Rad CFX96 system was used to acquire qPCR data
Data analysis	• IVIS images were analysed using the Living Image software (version 4.5.2) (Perkin Elmer).<br>• qPCR results were analysed using Microsoft Excel and GraphPad Prism (version 8)<br>- Immunofluorescence images were analysed using FiJI (version 2.14.0)

For manuscripts utilizing custom algorithms or software that are central to the research but not yet described in published literature, software must be made available to editors and reviewers. We strongly encourage code deposition in a community repository (e.g. GitHub). See the Nature Portfolio guidelines for submitting code & software for further information.

Data

Policy information about availability of data

All manuscripts must include a data availability statement. This statement should provide the following information, where applicable:

- Accession codes, unique identifiers, or web links for publicly available datasets
- A description of any restrictions on data availability
- For clinical datasets or third party data, please ensure that the statement adheres to our policy

Numerical data corresponding to all graphs in the figures is provided in Supplementary Data File 1.

Research involving human participants, their data, or biological material

Policy information about studies with human participants or human data. See also policy information about sex, gender (identity/presentation), and sexual orientation and race, ethnicity and racism.

Reporting on sex and gender	*Use the terms sex (biological attribute) and gender (shaped by social and cultural circumstances) carefully in order to avoid confusing both terms. Indicate if findings apply to only one sex or gender; describe whether sex and gender were considered in study design; whether sex and/or gender was determined based on self-reporting or assigned and methods used. Provide in the source data disaggregated sex and gender data, where this information has been collected, and if consent has been obtained for sharing of individual-level data; provide overall numbers in this Reporting Summary. Please state if this information has not been collected. Report sex- and gender-based analyses where performed, justify reasons for lack of sex- and gender-based analysis.*
Reporting on race, ethnicity, or other socially relevant groupings	*Please specify the socially constructed or socially relevant categorization variable(s) used in your manuscript and explain why they were used. Please note that such variables should not be used as proxies for other socially constructed/relevant variables (for example, race or ethnicity should not be used as a proxy for socioeconomic status). Provide clear definitions of the relevant terms used, how they were provided (by the participants/respondents, the researchers, or third parties), and the method(s) used to classify people into the different categories (e.g. self-report, census or administrative data, social media data, etc.) Please provide details about how you controlled for confounding variables in your analyses.*
Population characteristics	*Describe the covariate-relevant population characteristics of the human research participants (e.g. age, genotypic information, past and current diagnosis and treatment categories). If you filled out the behavioural & social sciences study design questions and have nothing to add here, write "See above."*
Recruitment	*Describe how participants were recruited. Outline any potential self-selection bias or other biases that may be present and how these are likely to impact results.*
Ethics oversight	*Identify the organization(s) that approved the study protocol.*

Note that full information on the approval of the study protocol must also be provided in the manuscript.

Field-specific reporting

Please select the one below that is the best fit for your research. If you are not sure, read the appropriate sections before making your selection.

☒ Life sciences ☐ Behavioural & social sciences ☐ Ecological, evolutionary & environmental sciences

For a reference copy of the document with all sections, see nature.com/documents/nr-reporting-summary-flat.pdf

Life sciences study design

All studies must disclose on these points even when the disclosure is negative.

Sample size	For all experiments, sample size was chosen to ensure accurate, reproducible results. Mouse IVIS imaging experiments involved a minimum of 3 mice per group, with data obtained from a minimum of 2 separate litters and images. Mouse tissue for qRT-PCR experiments was dissected from at least 3 mice. This study did not involve any external intervention to the mice and the animals used were of equivalent ages and housed identically, therefore, we deemed these sample sizes enough to produce reproducible results whilst minimising the number of animals sacrificed, in accordance with the NC3Rs.<br><br>Cell culture experiments were performed in a minimum of triplicate. The cells and culture conditions used was kept consistent across experiments and so we determined that this was enough to ensure accurate, reproducible results and perform statistical analysis. RT-qPCR experiments were performed in technical triplicate to account for pipetting errors.
Data exclusions	The only data excluded from this study were a small number of technical replicates from RT-qPCR analysis. In this case, each biological sample was run in triplicate wells, should one well deviate substantially from the other two wells then it was removed from the analysis and a mean determined from the remaining two samples. Biological replicates were not excluded.
Replication	All experiments were performed in replicate to confirm the reproducibility of the findings, in all cases this was successful.<br>To maintain the reproducibility of findings, all animals used in this study were maintained using the same conditions and housed in the same animal holding room. This means that diet, microbiota, light/dark cycles, temperature and cage enrichment will all be equivalent. All animals used in this study were between 3-16 weeks old, and within individual experiments the age difference never exceeded 1 week. Other experimental parameters such as time between D-Luc exposure and imaging for IVIS experiments and conditions of RNA extraction from tissues were determined initially and maintained across experimental replicates to ensure consistency.<br>Similarly, for cell culture experiments, the culture conditions and passage number of the cells used was maintained as consistent as possible throughout.
Randomization	For the animal experiments performed in this study, randomisation was not relevant as the tested variable was the genotype of the animal. Likewise, for cell culture experiments involving drug tested vs vehicle tested samples the cells were split from the same initial vial, grown in equivalent conditions prior to treatment and, with the exception of the treatment, all conditions during treatment were identical for both samples. Therefore, randomization was not required.

Blinding	For mouse adult/embryo experiments involving mixed genotypes, researchers were blinded to the genotypes of the animals involved during data collection. For bioluminescence drug treatment experiments involving embryonic stem cells, researchers were not blinded, this is because the location of the control sample needs to be known to determine that the imaging is successful and as an essential part of the data analysis.

Reporting for specific materials, systems and methods

We require information from authors about some types of materials, experimental systems and methods used in many studies. Here, indicate whether each material, system or method listed is relevant to your study. If you are not sure if a list item applies to your research, read the appropriate section before selecting a response.

Materials & experimental systems

n/a	Involved in the study	
☐	☒	Antibodies
☐	☒	Eukaryotic cell lines
☒	☐	Palaeontology and archaeology
☐	☒	Animals and other organisms
☒	☐	Clinical data
☒	☐	Dual use research of concern
☒	☐	Plants

Methods

n/a	Involved in the study	
☒	☐	ChIP-seq
☐	☒	Flow cytometry
☒	☐	MRI-based neuroimaging

Antibodies

Antibodies used	- Firefly luciferase: Rabbit monoclonal antibody [clone EPR17790] to Firefly Luciferase from Abcam (Catalogue Number ab185924).<br>- Alexa Flour-488 conjugated secondary antibody (Invitrogen 1874771)<br>- anti-CD45-PerCP-Cy5.5 antibody (clone 30-F11, Cat No. 103112, Biolegend)
Validation	- Firefly luciferase: as shown in the datasheet for ab185924, the antibody was shown to specifically detect firefly luciferase in HEK293T cells transfected with a vector containing firefly luciferase cDNA, and no signal was detected with empty vector. We further confirm this by testing different dilutions and using WT mouse gut and lung tissues as negative controls in order to determine the appropriate background signal.<br>-Fluorescent-conjugated antibodies were validated by the manufacturer in accordance with their datasheet and websites

Eukaryotic cell lines

Policy information about cell lines and Sex and Gender in Research

Cell line source(s)	C57BL/6 knock-in mESCs clones and Bruce4 parental wild-type mESCs were received from OzGene, Australia.
Authentication	The commercial provider of the cell lines made their in house authentication including karyotyping and transgenic KI genotyping. We further confirm the identity of KI clones by genotyping PCR and qPCR to assess firefly luciferase expression.
Mycoplasma contamination	All cell lines used in this study tested negative for mycoplasma.
Commonly misidentified lines<br>(See ICLAC register)	n/a

Animals and other research organisms

Policy information about studies involving animals; ARRIVE guidelines recommended for reporting animal research, and Sex and Gender in Research

Laboratory animals	Cyp1a1-Fluc (referred to as Cyp1a1F) knock in mouse line was generated by OzGene, Australia on a C57BL/6 strain background.
Wild animals	No wild animals were involved in this study
Reporting on sex	Both male and female mice were used in all in vivo experiments with the aim to have equal sex replicate numbers if possible. We limit to a single sex within a single experiment to compare treated to untreated.
Field-collected samples	No field-collected samples were involved in this study
Ethics oversight	All animal procedures were performed in accordance with the British Home Office Animal (Scientific Procedures) Act 1986 . The mouse work was approved by the Imperial College AWERB committee and performed under a UK Home Office Project License and Personal Licenses.

Note that full information on the approval of the study protocol must also be provided in the manuscript.

Flow Cytometry

Plots

Confirm that:

☒ The axis labels state the marker and fluorochrome used (e.g. CD4-FITC).

☒ The axis scales are clearly visible. Include numbers along axes only for bottom left plot of group (a 'group' is an analysis of identical markers).

☒ All plots are contour plots with outliers or pseudocolor plots.

☒ A numerical value for number of cells or percentage (with statistics) is provided.

Methodology

Sample preparation	To generate a single cell suspension enriched for intestinal intraepithelial lymphocytes, small intestine tissue was dissected and disassociated with 100 mM DTT, prior to lymphocyte purification using a Percoll gradient. A detailed procedure is included in the Methods section. Cells were then stained with antibody at 4 degrees for 1 hour, washed and stained with DAPI for cell viability 5 minutes before taking to the flow cytometer.
Instrument	BD FACSAria™ III
Software	BD FACSDiva™ Software for sample acquisition and data recording.<br>FlowJo for data analysis and plotting. Adobe Illustrator for plot refining and layout editing.
Cell population abundance	From the total cell suspension sample, after gating on lymphocytes, single cells and live cells, the CD45 positive population was over 90% (91.5 to 96.6%) for all the samples. This indicates that our intestinal intraepithelial lymphocyte preparation was enriched for immune cells as labeled by the CD45 surface marker.
Gating strategy	The first gate was SSC-A and FSC-A, where lymphocyte population located at the lower SSC-A (50K) and medium FSC-A (100-150K) was gated. Then single cells were gated using FSC-H and FSC-A using a diagonal gate from 50-150K for both axes. From this population, DAPI and FSC-A were used to select live cells by gating the population located around 10^2 for DAPI and 100-200K for FSC-A. Finally, CD45-PerCP-Cy5.5 vs FSC-A was used to gate CD45 positive cells by gating around 10^3 to 10^4 corresponding to 100-200K for FSC-A.

☒ Tick this box to confirm that a figure exemplifying the gating strategy is provided in the Supplementary Information.
